# Repurposing of sericin combined with dactolisib or vitamin D to combat non-small lung cancer cells through computational and biological investigations

**DOI:** 10.1038/s41598-024-76947-0

**Published:** 2024-11-07

**Authors:** Maged W. Helmy, Mariam H. Youssef, Imane Yamari, Alaa Amr, Farouzia I. Moussa, Abeer El Wakil, Samir Chtita, Lamia M. El-Samad, Mohamed A. Hassan

**Affiliations:** 1 Pharmacology and Toxicology Department, Faculty of Pharmacy, Damanhour University, 22511 Damanhour, Egypt; 2 Department of Zoology, Faculty of Science, Alexandria University, Alexandria, Egypt; 3 Laboratory of Analytical and Molecular Chemistry, Faculty of Sciences Ben M’Sik, Hassan II University of Casablanca, P. O. Box 7955, Casablanca, Morocco; 4 Department of Biological and Geological Sciences, Faculty of Education, Alexandria University, Alexandria, Egypt; 5 Protein Research Department, Genetic Engineering and Biotechnology Research Institute (GEBRI), City of Scientific Research and Technological Applications (SRTA-City), New Borg El-Arab City, 21934 Alexandria Egypt

**Keywords:** Sericin, Dactolisib, Vitamin D, Lung cancer, Non-small lung cancer cells, Nuclear factor Kappa B (NF-κB), Phospho-akt (p-AKT), Cyclin D1, Vascular endothelial growth factor 1 (VEGF1), In silico studies, Protein-protein interaction, Biochemistry, Cancer, Computational biology and bioinformatics, Drug discovery

## Abstract

This study aims to repurpose sericin in combating non-small lung cancer cells (A549 and H460) by combining it with dactolisib or vitamin D to reduce the dose of dactolisib and boost the anticancer effectiveness of dactolisib and vitamin D. Therefore, the binding affinities of individual and combined drugs were examined using in silico and protein-protein interaction studies, targeting NF-κB, Cyclin D1, p-AKT, and VEGF1 proteins. The findings manifested remarkable affinities for combinatorial drugs compared to individual compounds. To substantiate these findings, the combined IC_50_ for each combination (sericin + dactolisib and sericin + vitamin D) were determined, reporting 31.9 and 41.8 µg/ml, respectively, against A549 cells and 47.9 and 55.3 µg/ml, respectively, against H460 cells. Furthermore, combination indices were assessed to lower the doses of each drug. Interestingly, in vitro results exhibited marked diminutions in NF-κB, Cyclin D1, p-AKT, and VEGF1 after treatment with sericin + dactolisib and sericin + vitamin D compared to control lung cancer cells and those treated with a single drug. Moreover, A549 and H460 cells treated with both combinations demonstrated augmented caspase-3 levels, implying substantial apoptotic activity. Altogether, these results accentuated the prospective implementation of sericin in combination with dactolisib and vitamin D at low doses to preclude lung cancer cell proliferation.

**Supplementary Information:**

The online version contains supplementary material available at 10.1038/s41598-024-76947-0.

## Introduction

Cancer was defined as the most pervasive determinant of mortality in 2023, and the most prevalent types of cancer globally include lung, breast, colorectum, and prostate cancers, respectively^1^. Cancer arises from epigenetic and genetic mutations that interfere with normal cellular growth, resulting in uncontrollable proliferation. This instigates cancer cells to evade cell cycle arrest and apoptosis, leading to their metastasis into adjacent tissues^2,3^. It has been reported that among various types of cancer, lung cancer is the predominant cause of cancer mortality, which is classified into non-small cell lung cancer (NSCLC) and small cell lung cancer (SCLC)^4–6^. Following GLOBOCAN 2022, it has been estimated about 1.8 million deaths and 2.5 million new cases due to lung cancer^7^. Furthermore, According to recent statistics on lung cancer predominance, NSCLC represents 85%, while only 15% belongs to SCLC^8^. The management of lung cancer remains a clinical challenge, given its considerable morbidity and mortality rates. Therefore, various therapeutic strategies have been implemented for governing lung cancer, including chemotherapy, stem cell transplant, surgery, radiation therapy, immunotherapy, precision medicine, targeted drugs, and palliative care^9^. Chemotherapeutic compounds have been broadly utilized for combating lung cancers, which fundamentally rely on the orchestration of signaling pathways; however, lung cancer reveals critical impediment to chemotherapy^10,11^. It is thus crucial to seek out more effective, selective chemotherapeutic compounds that are less costly, have fewer side effects, and exhibit minimum levels of disease resistance.

Among effective anticancer chemotherapies, dactolisib is an imidazoquinoline compound that has demonstrated significant anticancer activity, exerting dual inhibitory properties against phosphoinositide 3-kinase (PI3K) and the mammalian target of rapamycin complex 1 (mTORC1)/2^10,12^. It has been demonstrated to preclude the up-regulation of p-AKT, which is mediated by mTORC2. This inhibitory performance proposes the potential application of dactolisib to treat various types of tumors. In addition to its suppression of p-AKT, it can hamper Akt activation, highlighting the dual inhibition of PI3K and mTORC2^10^. This dual inhibition suggests that dactolisib may exert its anticancer effects through multiple pathways. Furthermore, dactolisib plays a pivotal role in autophagy activation, implying another mechanism of dactolisib in disintegrating cancer cells^13,14^. However, it has been reported that such an anticancer compound has critical disadvantages due to its failure to select mTOR and its reaction with DNA-dependent protein kinase, engendering unanticipated pernicious effects on normal cells^15^.

A previous investigation reported the anticancer potency of dactolisib in vitro against several cell lines; however, in vivo studies demonstrated the emergence of alopecia, hyperglycemia, and liver cytolysis in mice^16^. Furthermore, the administration of dactolisib alone could provoke disease stagnation; however, the combination of dactolisib with other anticancer agents could effectively boost their anticancer properties^10^. It is therefore warranted to reduce the doses of dactolisib by combining it with other anticancer compounds, providing high compatibility with normal cells.

To debilitate these flaws, natural products have emerged as a noteworthy supplementary management option against this intractable disease. Silk sericin is defined as a bioactive protein purified from silkworm cocoons of *Bombyx mori*^17,18^. Given exceptional biological and chemical properties of sericin, it has been implemented in numerous applications, including food and medical biotechnology^19–23^. Of particular interest, sericin exhibited inherent bioactivities, including antioxidant, antibacterial, and anti-inflammatory attributes^18,19,22,24^. As an anticancer compound, recent investigations emphasized the potency of sericin in inducing apoptosis of cancer cells^25^.

Another natural anticancer compound is vitamin D, which was characterized as a secosteroid prohormone, playing a key role in calcium and phosphorus homeostasis and bone metabolism^26^. Recently, it was manifested that vitamin D hampers the propagation of malignant cells and instigates inflammatory reactions^27^. Importantly, previous studies linked vitamin D deficiency to tumor progression and demonstrated its ability to reinforce cancer cells apoptosis^28,29^. Nevertheless, the most effective amount of vitamin D therapy for reducing or treating cancer risk remains unknown, in addition to the development of resistant malignant cells^30^. We thus presume that sericin could combine with dactolisib or vitamin D to diminish the dose of dactolisib and enhance the anticancer efficacy of dactolisib or vitamin D against non-small lung cancer cells (A549 and H460). To examine our assumption, we performed in silico investigations to evaluate the competency of all individual drugs and sericin combined with dactolisib or vitamin D targeting Nuclear Factor Kappa B (NF-κB), Cyclin D1, phospho-Akt (p-AKT), and vascular endothelial growth factor 1 (VEGF1) proteins. To verify the in silico findings, comprehensive in vitro studies against H460 and A549 cells were conducted after determining the IC_50_ of each drug and combination indices (Cls) to reduce the doses of each combination.

## Materials and methods

### Chemicals

Sericin powder from the silkworm *Bombyx mori* (Cat. No. S5201-5G) was supplied by Sigma-Aldrich (St. Louis, USA). Dactolisib BEZ-235 (Cat. No. S1009) was purchased from Selleckchem (Houston, TX, USA). Vitamin D oil Pharmaprim oral drops solution (80 IU/drop) was provided by Pharmaprim AB (Svärdvägen 3B, Sweden). Dimethyl sulfoxide (DMSO), fetal bovine serum (FBS), and 3-(4,5-dimethylthiazol-2-yl)-2,5-diphenyltetrazolium bromide (MTT) were procured from Sigma-Aldrich (St. Louis, USA). Trypsin, Dulbecco’s modified eagle medium (DMEM), phosphate buffer saline, and penicillin/streptomycin antibiotic mixtures were obtained from Lonza^®^ (Basel, Switzerland). RIPA cell lysis buffer (Cat. No. 89900), containing 25 mM Tris-HCl pH 7.6, 150 mM NaCl, 1% NP-40, 1% sodium deoxycholate, and 0.1% SDS was purchased from Thermo Scientific, USA. A stock solution of dactolisib was prepared at a concentration of 25 µM by dissolving in DMSO before being stored at − 20 °C.

### In silico studies

In this study, we utilized molecular docking simulations to explore interactions between selected compounds and proteins, focusing on both protein-ligand and protein-protein interactions. Molecular docking is a pivotal in deciphering molecular biological processes, offering a predictive framework for delineating binding modes and affinities in small compound-protein interactions^31–34^. This approach facilitates the rational design of pharmaceutical agents by identifying molecules with favorable binding interactions^35^. Simultaneously, molecular docking in protein-protein interactions is crucial for unraveling cellular signaling intricacies, providing critical insights into cellular function regulation^36^. The dual approach of investigating both interactions provides a comprehensive understanding of molecular binding, empowering our study with a computational tool to predict, analyze, and comprehend interactions, contributing to drug discovery and fundamental research^37^.

To explore protein-ligand interactions, vitamin D (https://pubchem.ncbi.nlm.nih.gov/compound/5280795) and dactolisib (https://pubchem.ncbi.nlm.nih.gov/compound/11977753) were selected from the PubChem Database (https://pubchem.ncbi.nlm.nih.gov/), and their structures were subsequently optimized using Avogadro software (https://avogadro.cc/)^38^. Four target proteins (NF-κB, Cyclin D1, p-AKT, and VEGF1) were selected for docking studies. Their structures obtained from the RCSB Protein Data Bank (https://www.rcsb.org/)^39^ with PDB IDs 4G3D, 2W96, 3O96, and 1FLT were refined using Swiss PDB Viewer (https://spdbv.unil.ch/selmenu_menu.html) to ensure accurate structural representation^40^. A detailed protein preparation protocol was implemented to create an optimal environment for molecular docking. Water molecules were removed, polar hydrogen atoms were added to the protein structures, and Kollman charges were assigned to ensure an accurate representation of the molecular electrostatic potential. Subsequently, the grid box for the docking simulations was defined, determining the search space for the ligand within the protein’s binding site. The dimensions of the grid box are shown in Table 1. AutoDock Vina (https://vina.scripps.edu/)^41^ was employed to perform docking simulations, allowing for the exploration of diverse binding modes and affinities between the compounds and the target proteins^42^. The resulting interactions were visualized using BIOVIA Discovery Studio Visualizer (https://discover.3ds.com/discovery-studio-visualizer-download) providing detailed molecular interaction insights^43,44^.

**Table 1:** Protein’s active site coordinates.

Protein	Size (Å)	Center (Å)
4G3D	X = 40; Y = 40; Z = 40	X = 27.33
		Y = 3.32
		Z = 93.13
2W96	X = 40; Y = 40; Z = 40	X =−3.94
		Y = 14.75
		Z = 44.90
3O96	X = 40; Y = 40; Z = 40	X = 11.12
		Y = 0.58
		Z = 29.12
1FLT	X = 40; Y = 40; Z = 40	X = 14.12
		Y = 1.47
		Z = 19.54

Transitioning to the investigation of protein-protein interactions, the 3D structure of sericin (PDB ID: 3ULT) is docked into the active site with the previously mentioned four proteins. We utilized the HADDOCK 2.4 (https://rascar.science.uu.nl/haddock2.4/) a web server that facilitates protein-protein docking studies through an intuitive interface^45,46^, employing an iterative and clustering algorithm to consider molecular flexibility. Its significance lies in providing accurate predictions, essential for understanding dynamic protein interactions.

We used the previously prepared protein with refinement through energy minimization using the Swiss PDB Viewer^40^. To guide the docking simulation, the active binding sites for each protein were predicted using the MetaPPISP tool (https://pipe.rcc.fsu.edu/meta-ppisp.html)^47^ as presented in Table 2. Subsequently, we submitted the docking studies to the HADDOCK 2.4 web server, leveraging its clustering algorithm. The more negative HADDOCK scores are indicative of energetically stable complexes, suggesting strong binding affinities between the molecules involved. The extracted data from the HADDOCK scoring output for docked sericin against the investigated proteins included five key HADDOCK features: HADDOCK score, RMSD (Root Mean Square Deviation), Z-score, Van Der Waals forces, and electrostatic energy (https://www.bonvinlab.org/education/HADDOCK24/HADDOCK24-protein-protein-basic/#introduction).

The outcomes were meticulously analyzed using Discovery Studio Visualizer software (https://discover.3ds.com/discovery-studio-visualizer-download), providing comprehensive visualization and potential binding modes of the sericin and target protein complexes. Furthermore, we performed residue interaction analysis on the obtained complexes from the HADDOCK 2.4 web server using the molecular data resource of PDBsum (https://www.ebi.ac.uk/thornton-srv/databases/pdbsum/)^48^. This analysis was aimed at identifying all types of non-covalent interactions at the atomic level within the studied protein-protein complexes. Specifically, the focus was on understanding the strength of sericin binding to the lateral region of each target protein. This comprehensive methodology aimed to unveil intricate details of protein-protein interactions, contributing valuable insights into the functional relationship between sericin and target proteins.

To extend our investigation, we also ran a docking simulation to explore the molecular interactions involving sericin, the dactolisib compound, and additional key proteins (NF-κB, CyclinD1, p-AKT, and VEGF). The methodological approach incorporated precise parameterization within the HADDOCK server, considering the individual molecular structures and active sites of the studied proteins. The iterative docking procedure yielded detailed insights into energetically favorable conformations and binding affinities. In parallel, our study expanded to explore the molecular intricacies of the sericin and vitamin D compound interaction. These findings contribute to our understanding of the versatile interactions of sericin in diverse biological contexts, providing insights for potential therapeutic interventions.

**Table 2:** Binding active sites for each studied receptor.

Structure	Chain ID	Active site residues
Sericin (3ULT)	B	34, 35, 49, 50, 52, 61, 62, 63, 64, 66, 75, 76, 77, 78, 80, 90, 92, 94, 106, 107, 108
NF-κB (4G3D)	D	401, 407, 408, 409, 410, 411, 412, 413, 414, 415, 416, 430, 469, 471, 473, 514, 519, 534, 536, 557, 558, 559, 560, 563, 594, 597, 601, 606
Cyclin D1 (2W96)	A	6, 7, 8, 9, 10, 11, 12, 13, 25, 26, 27, 29, 30, 34, 113, 153, 155, 159, 162, 163, 166, 167, 213, 217, 220, 221, 222, 224, 225, 226, 227, 228, 231, 232
p-AKT (3O96)	A	155, 156, 157, 229, 231, 233, 234, 236, 237, 240, 241, 242, 243, 244, 272, 274, 278, 279, 292, 293, 294, 298, 345, 346, 347
VEGF1 (1FLT)	W	12, 13, 14, 15, 16, 17, 18, 19, 20, 21, 22, 23, 24, 25, 27, 29, 57, 58, 59, 60, 62, 63

### Cell lines and culture conditions

The human non-small-cell lung cancer cell lines (H460 and A549) were supplied by the American Type Culture Collection (ATCC, USA). A549 and H460 cells were nurtured in DMEM supplemented with 10% FBS, 100 U/ml penicillin, and 100 µg/ml streptomycin and then incubated in a humidified CO_2_ incubator with 5% CO_2_ at 37 ºC. Briefly, at 80% confluence, cells were harvested using 0.25% (w/v) trypsin solution, followed by subculturing into T-75 flasks or 96-well plates according to the experiment. The investigations were optimized to determine the most suitable seeding densities of cells. Concerning the 96-well plate, 1 × 10^3^, 15 × 10^3^, 20 × 10^3^, and 30 × 10^3^ cells were seeded into individual wells in a total volume of 200 µl. Concerning T-75 culture flasks, 75 × 10^4^, 15 × 10^5^, 20 × 10^5^, and 30 × 10^5^ cells were seeded in a total volume of 10 ml. Each experiment was conducted at least in triplicate, and cells were grown in a humidified CO_2_ incubator for 48 h. Following these experiments, 1 × 10^3^ cells/well and 2 × 10^5^ cells/flask were ascertained as initial seeding densities for further investigations.

### Growth inhibition assay

The growth inhibition of A549 and H460 cells in response to treatment with either sericin or sericin in combination with dactolisib or vitamin D was assessed following the MTT assay. To perform the test, cells were seeded in 96 well plates at a density of 4 × 10^3^ cells/well, followed by incubation in a CO_2_ incubator at 37 °C for 24 h. After aspiration of old media, 100 µl of treatment media were added to all wells, and different concentrations of sericin (280, 140, 70, 35, 17.5, and 8.75 µg/ml), dactolisib in DMSO (1.89, 0.94, 0.47, 0.23, 0.12, and 0.06 µg/ml), and/or vitamin D (19.23, 9.67, 4.81, 2.40, 1.20, and 0.6 µg/ml) were applied. Following incubation for a further 48 h, 20 µl of MTT reagent (5 mg/ml) was added to the wells and incubated for 4 h in a CO_2_ incubator^49,50^. After centrifuging the plates at 150 rpm for 10 min, the MTT reagent was aspirated. Following this, 150 µl of DMSO was applied to each well to dissolve the formazan crystals, and the absorbance was then determined at 590 nm wave length with a reference filter of 620 nm by means of a microplate reader (Bio-Rad, USA)^50–52^. The MTT assay was conducted in three independent replicates, and cell viability was estimated as percentages in relation to the untreated control wells. The half-maximal inhibitory concentrations (IC_50_) were calculated using the CompuSyn software (CompuSyn, Inc., version 1) following the previously published approach^53^.

### Drugs combination analysis

To evaluate the anticancer attributes of sericin and its combination with dactolisib or vitamin D against the A549 and H460 cells, the MTT assay was conducted as mentioned above. In this regard, cells were incubated with each drug alone and in combination, following the similar range of doses applied in the former study for 48 h before assessing the cytotoxicity. Accordingly, the Combination Index (CI) was quantified using the following equation to explore synergism or antagonism properties between the two drugs as described earlier^53,54^. The CIs < 1, = 1, and > 1 point to synergistic, additive, and antagonistic impacts, respectively. Moreover, the dose reduction index (DRI), the measurement of the fold decrease of individual agents when used in synergistic combinations to achieve a specified effect level in comparison to the doses of each drug alone, was evaluated by means of CompuSyn software^55^.

Combination index (Cl) = E (cA) E (DA) + E (cB) E (dB).

Where E (cA) points to the effect for drug (A) in a combination, E (cB) indicates the effect for drug (B) in combination, E (DA) refers to the effect for drug (A) alone, and E (dB) is the effect for drug (B) alone.

### Experimental design and treatment of A549 and H460 cells with drugs

Treatment groups were set out as follows: group I (vehicle only as vehicle control), group II [sericin (Ser), IC_50_ = 62.4 µg/ml], group III [dactolisib (Dac) IC_50_ = 0.6 µg/ml], group IV [vitamin D (VD) IC_50_ = 11.6 µg/ml], group V (Ser + Dac, at the same concentration of each drug alone), group VI (Ser + Dac, 31.9 mg/ml + 0.212 µg/ml, reduced synergistic doses of sericin and dactolisib as calculated following the DRI values using CompuSyn), group VII (Ser + VD at the same concentration of each drug alone), and group VIII (Ser + VD, 41.8 µg/ml + 3.49 µg/ml, reduced synergistic doses of sericin and dactolisib as calculated following the DRI values using CompuSyn).

For H460 cells, similar groups were designed with different doses as follows: group I (vehicle only as vehicle control), group II (Ser, IC_50_ = 83 µg/ml), group III (Dac, IC_50_ = 0.8 µg/ml), group IV (VD, IC_50_ = 17.5 µg/ml), group V (Ser + Dac, at the same concentration of each drug alone), group VI (Ser + Dac, 47.9 µg/ml + 0.314 µg/ml), group VII (Ser + VD at the same concentration of each drug alone), and group VIII (Ser + VD, 55.3 µg/ml + 4.62 µg/ml).

On day 0, equal numbers of A549 and H460 cells at a density of 2 × 10^5^ were seeded into 12 identical T-75 culture flasks, and cells were then allowed to adhere overnight. The following day (day 1), drugs were added to each flask according to the experimental design, while DMSO was added to the vehicle control group (I), and the flasks were incubated for 48 h. On day 3, cells were harvested using trypsin (0.25%), resuspended in PBS, and either used directly in downstream investigations or stored as aliquots at − 80 °C for further examinations. Each treatment was conducted in triplicate for three independent experiments.

### Preparation of cell lysate

To obtain cell lysates, the H460 and A549 cells exposed to different drugs and corresponding combinations were treated with RIPA cell lysis (Sigma-Aldrich, Germany) buffer following the manufacturer’s instructions. Briefly, 1 ml of RIPA buffer containing protease inhibitor cocktail was added to cell pellets, shaken gently for 15 min on ice, and then clarified by centrifugation at 14,000 ×g for 15 min. The supernatants were collected and stored at − 80 °C for subsequent assays.

### Evaluation of cancer biomarkers

Protein contents were determined by the bicinchoninic acid (BCA) method using the Pierce^®^ BCA protein assay kit. Additionally, the vitamin D receptor (VDR) was evaluated using a respective kit to explore the function of vitamin D as an anticancer agent. The phospho-Akt (p-Akt), phospho-nuclear factor kappa-light-chain enhancer of activated B cells (phospho-NF-κB), Cyclin D1, and vascular endothelial growth factor (VEGF) were assessed in the A549 and H460 cellular lysates of different treated groups using sandwich enzyme-linked immunosorbent assay (ELISA) kits as presented in Table S1. Values were normalized to their corresponding cellular total protein and presented as means ± SD of three independent experiments.

On the other hand, a colorimetric cysteine-requiring Aspartate protease (caspase-3 activity) assay kit shown in Table S1 was utilized to assess caspase-3 in cell lysates through the determination of a p-nitroaniline as a result of a peptide substrate (Ac-DEVD-pNA) hydrolysis by caspase-3. The concentration of the cleaved moiety was calculated from a calibration curve with absorbance at 405 nm using a microtitre plate reader. The assay was performed in three independent replicates, and the results are shown as means ± SD.

### Statistical analysis

All biochemical assays were conducted in triplicate for three independent experiments. The statistical analyses of the data were executed employing Statistical Product and Service Solutions (SPSS), version 25 (IBM Software, Inc., Chicago, IL, USA) and the GraphPad Prism^®^ software package, version 8 (GraphPad Software Inc., CA, USA). The data underwent normal distribution following a Kolmogorov-Smirnov normality test. Then, the normally distributed data were analyzed by means of one-way analysis of variance (ANOVA) with Tukey’s test for multiple comparisons between groups as the post-hoc test. All values are shown as mean ± SD, and they were considered significant at *p* ≤ 0.05.

## Results and discussion

### Protein-ligand interactions

In the conducted docking simulation, the binding affinities between two compounds, vitamin D and dactolisib, and four distinct protein structures (NF-κB, CyclinD1, p-AKT, and VEGF1) were assessed based on energy scores measured in Kcal/mol as illustrated in Table 3. Notably, dactolisib exhibited a consistently lower energy score compared to vitamin D in three out of the four proteins, suggesting a potentially stronger binding affinity. Specifically, dactolisib demonstrated superior binding in interactions with Cyclin D1, indicating a significant advantage in this context. However, vitamin D displayed a lower score in the case of p-AKT, hinting at a potentially stronger binding affinity compared to dactolisib. These findings provide valuable insights into the relative binding strengths of the two compounds across different protein structures, highlighting their potential implications for biological interactions.

**Table 3:** Binding energies of dactolisib and vitamin D into the active sites of the studied compounds.

Compounds	NF-κB	Cyclin D1	p-AKT	VEGF1
	Scores kcal/mol			
Vitamin D	−7.6	−6.5	−9.9	−6.2
Dactolisib	−7.9	−8.5	−13.4	−7.2

**Fig. 1:**
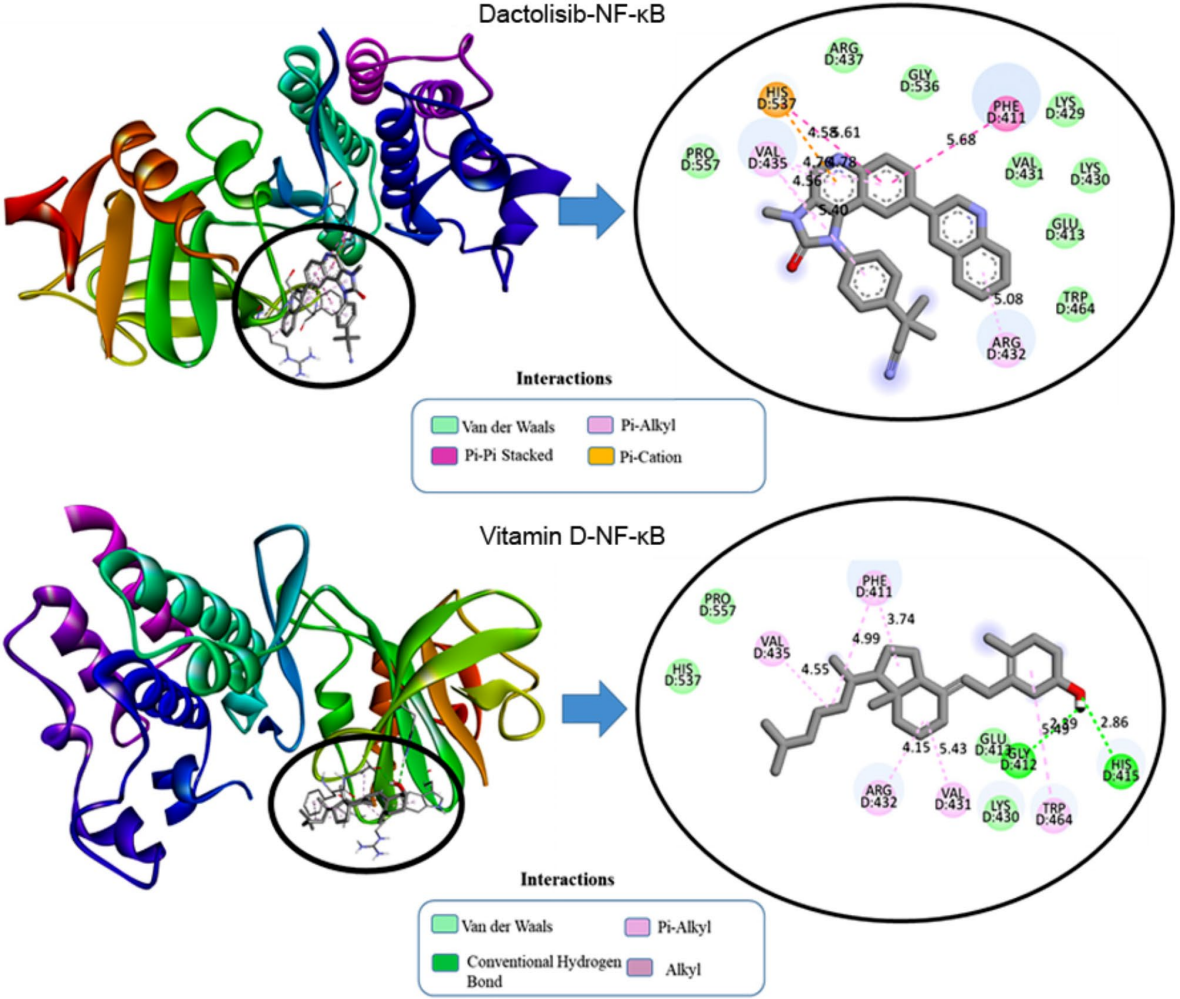
2D and 3D visualization of molecular interaction of dactolisib and vitamin D into the active site of NF-κB.

In the context of the docking simulation between compounds and protein structures, specific interactions were observed, revealing the molecular basis of binding between the ligands (vitamin D and dactolisib) and the target proteins (NF-κB, CyclinD1, p-AKT, and VEGF1). Considering the interaction of dactolisib with NF-κB, dactolisib exhibited an electrostatic interaction with histidine at position 537 (D: HIS537) through Pi-Cation at a distance of 4.59 Å as delineated in Fig. 1. Hydrophobic interactions were also observed, including Pi-Pi Stacked interactions with phenylalanine at position 411 (D: PHE411) and multiple Pi-Alkyl interactions with valine at position 435 (D: VAL435) and arginine at position 432 (D: ARG432). These interactions occurred at distances ranging from 4.59 Å to 5.62 Å. The engagement of dactolisib with NF-κB involves a combination of electrostatic and hydrophobic interactions, contributing to the overall stability of the ligand-protein complex.

**Fig. 2:**
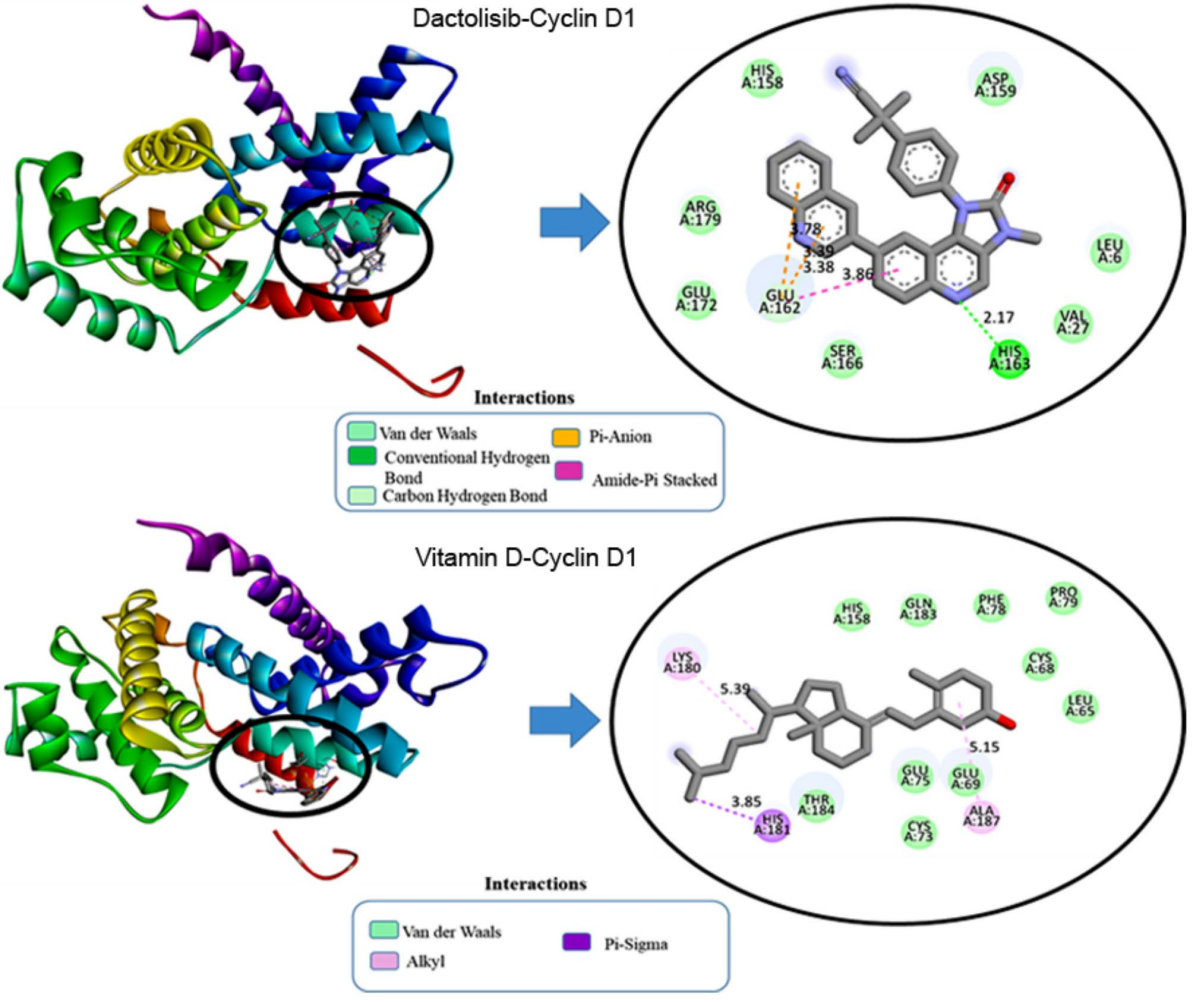
2D and 3D visualization of molecular interaction of dactolisib and vitamin D into the active site of Cyclin D1.

In the case of Cyclin D1, vitamin D demonstrated a hydrophobic interaction with histidine at position 181 (A: HIS181) through both Pi-Sigma and Pi-Orbitals at a distance of 3.86 Å as portrayed in Fig. 2. Additionally, hydrophobic interactions were observed between vitamin D and lysine at position 180 (A: LYS180) and alanine at position 187 (A: ALA187) through alkyl interactions at distances of 5.39 Å and 5.15 Å, respectively. On the other hand, dactolisib exhibited distinct interactions, forming a hydrogen bond with histidine at position 163 (A: HIS163) at a distance of 2.18 Å, while also engaging in a hydrogen bond with the carbonyl group of glutamate at position 162 (A: GLU162) at a distance of 3.38 Å. Electrostatic interactions were observed between dactolisib and glutamate at position 162 (A: GLU162) through Pi-Anion interactions at distances of 3.39 Å and 3.78 Å. Additionally, hydrophobic interactions were noted between dactolisib and various residues, including a Pi-Pi T-shaped interaction at a distance of 5.99 Å. These interactions collectively contribute to the stabilization of the ligand within the binding pocket of Cyclin D1, providing insights into the molecular mechanism of binding.

**Fig. 3:**
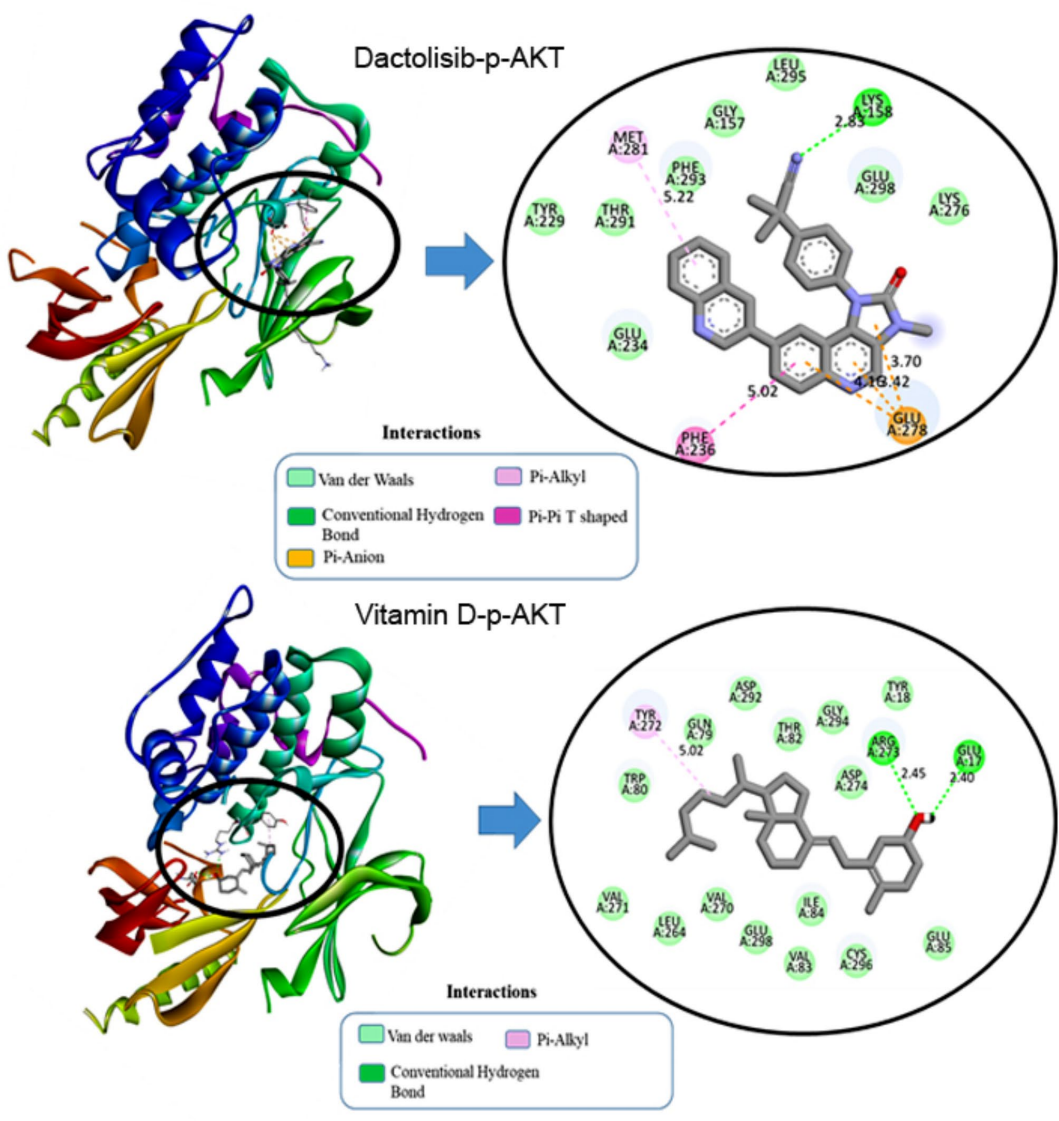
2D and 3D visualization of molecular interaction of dactolisib and vitamin D into the active site of p-AKT.

For the interaction of dactolisib with p-AKT in Fig. 3, the ligand forms a conventional hydrogen bond with LYS268 at a distance of 2.85 Å and a carbon-hydrogen bond with TYR272 at 3.39 Å. Electrostatic interactions include a Pi-Cation interaction with LYS268 at 4.25 Å. Hydrophobic interactions involve Pi-Sigma interactions with VAL270 at 3.82 Å and multiple Pi-Pi Stacked interactions with TRP80, with distances ranging from 3.88 Å to 4.99 Å. Additionally, Pi-Alkyl interactions are noted with VAL270, LEU264, and LYS268, with distances between 3.59 Å and 5.42 Å. These interactions contribute to the stabilization of dactolisib within the binding pocket, underscoring its robustness as a ligand. Vitamin D also forms a conventional hydrogen bond with TYR272 at 2.23 Å and exhibits prominent hydrophobic interactions, including alkyl interactions with LEU264, LYS268, and VAL270 (distances ranging from 4.02 Å to 5.41 Å) and Pi-Alkyl interactions with TRP80 at 4.02 Å. These interactions highlight the role of hydrophobic forces in stabilizing Vitamin D3 within the binding site, enhancing its binding affinity to the target protein.

**Fig. 4:**
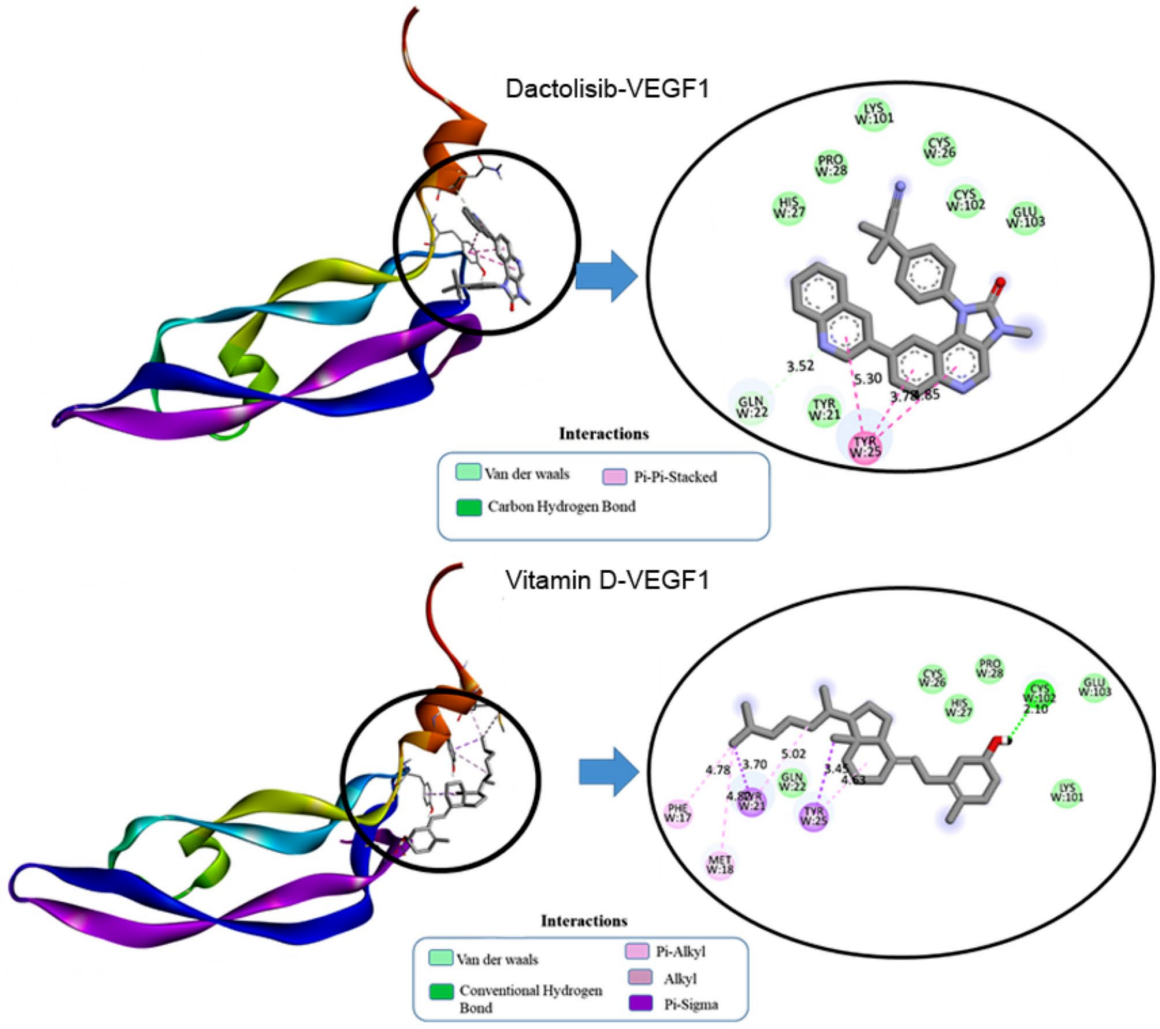
2D and 3D visualization of molecular interaction of dactolisib and vitamin D into the active site of VEGF1.

In VEGF1, dactolisib formed a carbon-hydrogen bond with glutamine at position 22 (W: GLN22) at a distance of 3.53 Å as depicted in Fig. 4. Hydrophobic interactions included Pi-Pi Stacked interactions with tyrosine at position 25 (W: TYR25) at distances ranging from 3.78 Å to 5.29 Å. Dactolisib also engaged in Pi-Sigma interactions with cysteine at position 102 (W: CYS102) and Pi-Alkyl interactions with tyrosine at positions 21 and 25 (W: TYR21, W: TYR25), with distances ranging from 2.11 Å to 5.02 Å. These interactions collectively contribute to the stability of the dactolisib-VEGF1 complex, providing a comprehensive understanding of the ligand’s binding mechanism within the protein’s active site.

### Protein-protein interaction

In a comprehensive examination of four distinct docking systems involving sericin (PDB ID: 3ULT) and various target proteins through the HADDOCK server, distinct binding characteristics and affinities have been uncovered as presented in Table 4.

In system 1 (NF-κB), the interaction between sericin and NF-κB demonstrated a robust binding affinity with a HADDOCK score of −96.8 ± 6.2, indicating a strong interaction. The RMSD value of 0.7 ± 0.4 suggested structural convergence among the generated clusters, while the Van der Waals and electrostatic energies highlighted favorable non-covalent interactions.

With regard to system 2 (Cyclin D1), it exhibited a strong binding affinity, reflected in the HADDOCK score of −93.5 ± 7.1 although the RMSD was slightly higher at 1.7 ± 1. The Van der Waals and electrostatic energies contributed significantly to the stabilization of the sericin-Cyclin D1 complex.

Considering System 3 (p-Akt), it demonstrated a potent binding affinity with a HADDOCK score of −109.6 ± 9.3, despite a higher RMSD of 9.3 ± 0.2, suggesting structural diversity among the generated clusters. The Van der Waals and electrostatic energies played crucial roles in stabilizing the complex.

In system 4 for (VEGF1) interaction, it revealed a moderate binding affinity with a HADDOCK score of −66 ± 8.5. The RMSD value of 2 ± 0.6 indicated a relatively convergent set of structures within the generated clusters. Both van der Waals and electrostatic energies contributed to the complex’s stabilization.

Comparatively, system 1 and system 2 displayed similar HADDOCK scores, indicating comparable binding affinities, while system 3 demonstrated a higher HADDOCK score, suggesting a potentially stronger interaction. System 4 showed a lower HADDOCK score, indicating a moderate binding affinity. The RMSD value varied with system 3, exhibiting greater structural diversity. Electrostatic and Van der Waals energies contributed to complex stabilization across all systems. Considering these findings, system 3, involving sericin and p-AKT, emerges as a promising candidate for further exploration due to its robust binding affinity.

For the other systems, the outcomes shown in Tables 5 and 6 revealed potential protein-protein interactions and binding sites, providing a comprehensive understanding of the molecular interaction when adding dactolisib or vitamin D compound to the complexes. And the interaction with p-AKT is more relevant in all cases.

**Fig. 5:**
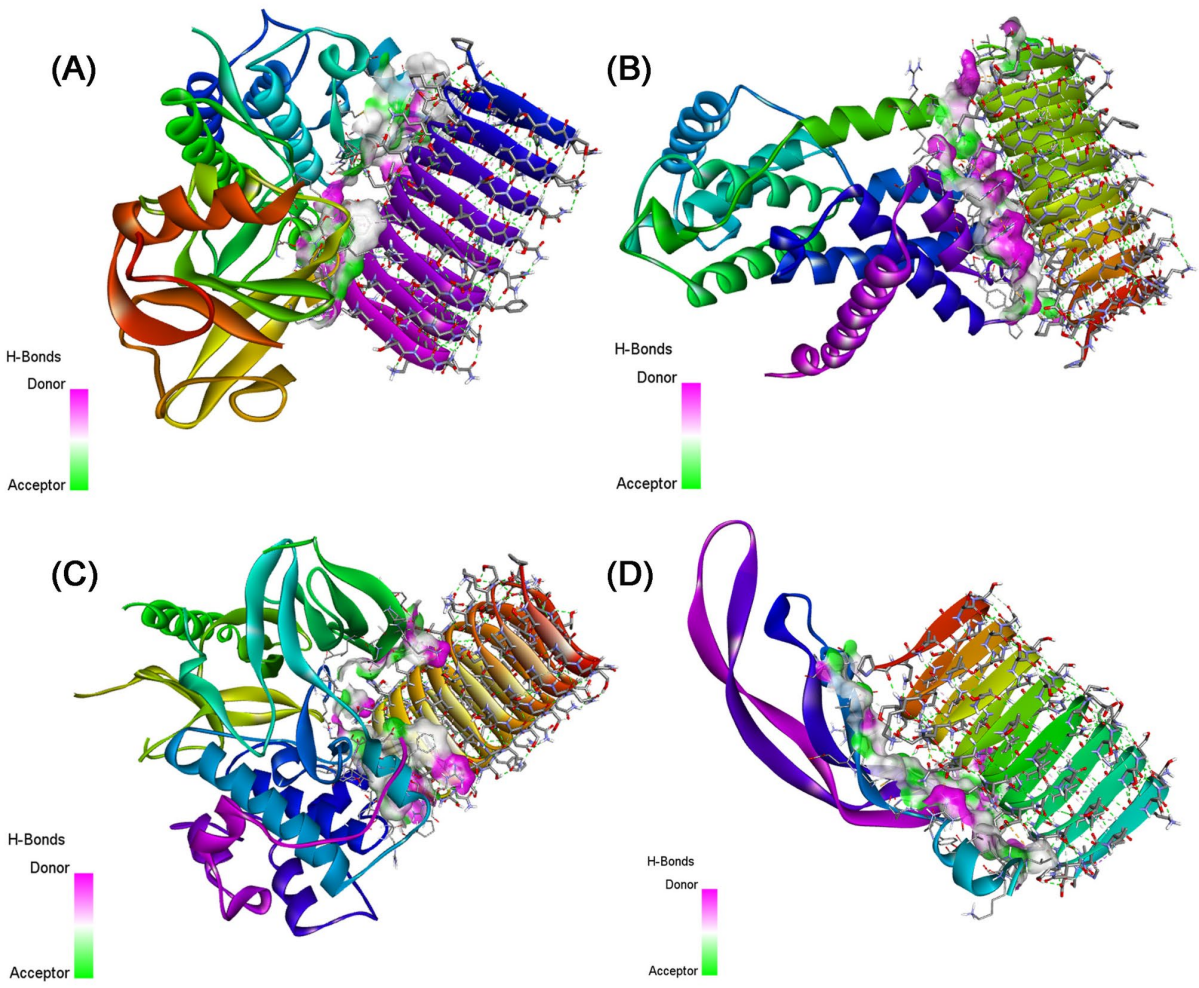
3D visualization of the non-covalent interactions made between (**A**) Sericin-NF-κB, (**B**) Sericin-Cyclin D1, (**C**) Sericin-p-AKT, and (**D**) Sericin-VEGF1.

**Fig. 6:**
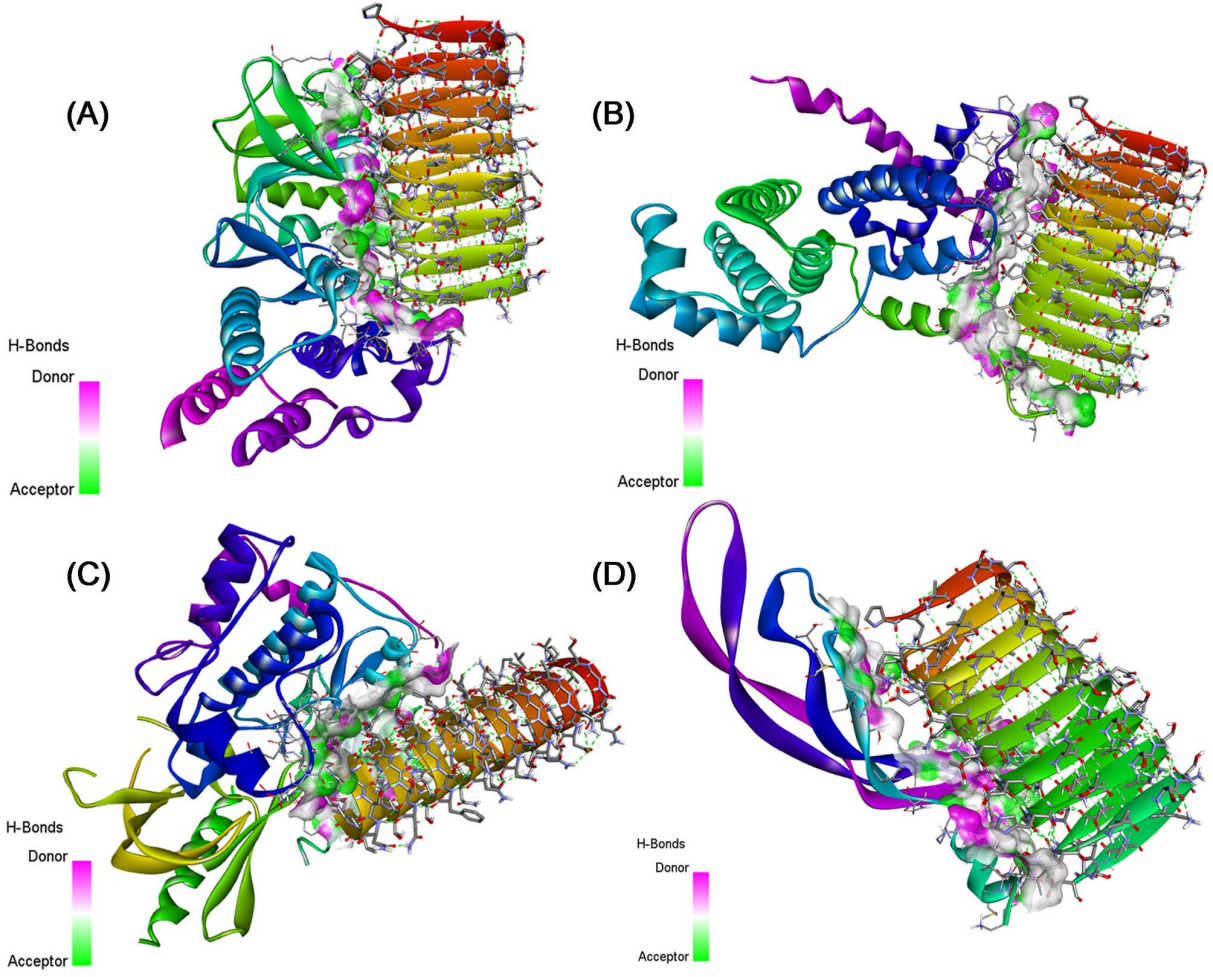
3D visualization of the non-covalent interactions made between (**A**) Sericin + Dactolisib-NF-κB, (**B**) Sericin + Dactolisib-Cyclin D1, (**C**) Sericin + Dactolisib-p-AKT, and (**D**) Sericin + Dactolisib-VEGF1.

**Fig. 7:**
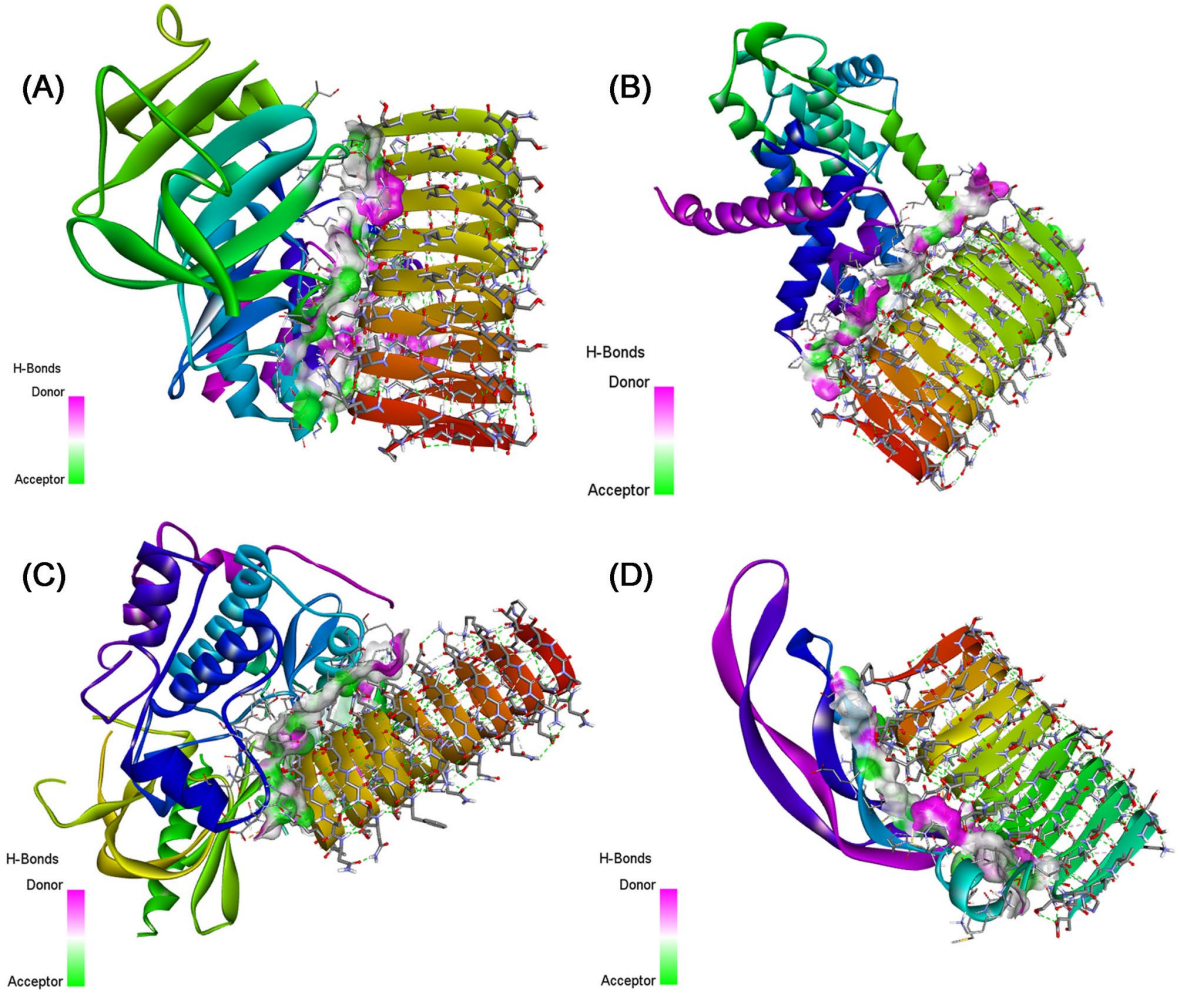
3D visualization of the non-covalent interactions made between (**A**) Sericin + Vitamin D-NF-κB, (**B**) Sericin + Vitamin D-Cyclin D1, (**C**) Sericin + Vitamin D-p-AKT, and (**D**) Sericin + Vitamin D-VEGF1.

Table S2 presents the details of the interacting residues, showing the non-covalent bonds within the studied complexes, including Van der Waals forces and hydrogen bonds. In addition, we used the EMBL-EBI molecular data repository to discern the list of interactions between residues across the protein-protein interface for the other the list of interactions between residues across the protein-protein interface for the other complexes as illustrated in Fig. S1–S8. These interactions were visualized using Discovery Studio software as shown in Figs. 5, 6, and 7.

**Table 4:** Docking parameters using the HADDOCK server for the studied complexes.

Complexes	Sericin-NF-κB	Sericin-Cyclin D1	Sericin-p-AKT	Sericin-VEGF1
Cluster number	Cluster 3	Cluster 4	Cluster 4	Cluster 7
HADDOCK score	−96.8 ± 6.2	−93.5 ± 7.1	−109.6 ± 9.3	−66.0 ± 8.5
RMSD from the overall lowest-energy structure	0.7 ± 0.4	1.7 ± 1.0	9.3 ± 0.2	2.0 ± 0.6
Van der Waals energy	−71.7 ± 4.4	−72.9 ± 16.9	−52.5 ± 7.7	−51.0 ± 7.0
Electrostatic energy	−223.4 ± 22.8	−278.2 ± 77.2	−417.8 ± 72.1	−154.1 ± 34.1
Z-score	−1.4	−1.9	−1.5	−1.5

**Table 5:** The HADDOCK parameters of interaction between sericin + dactolisib and the studied proteins.

Complexes	Sericin + Dactolisib-NF-κB	Sericin + Dactolisib-Cyclin D1	Sericin + Dactolisib-p-AKT	Sericin + Dactolisib-VEGF1
Cluster number	Cluster 5	Cluster 1	Cluster 4	Cluster 3
Haddock score	−89.9 ± 5.2	−92.4 ± 4.7	−117.2 ± 4.9	−71.6 ± 5.2
RMSD from the overall lowest-energy structure	0.6 ± 0.4	4.9 ± 0.2	0.4 ± 0.4	2.4 ± 1.4
Van der Waals energy	−52.1 ± 2.2	−60.8 ± 5.3	−86.4 ± 6.5	−59.6 ± 3.5
Electrostatic energy	−340.6 ± 34.0	−326.4 ± 17.0	−212.6 ± 15.3	−142.4 ± 37.7
Z-score	−1.4	−1.5	−1.8	−2.3

**Table 6:** The HADDOCK parameters of interaction between sericin + vitamin D and the studied proteins.

Complexes	Sericin + Vitamin D-NF-κB	Sericin + Vitamin D-Cyclin D1	Sericin + Vitamin D-p-AKT	Sericin + Vitamin D-VEGF1
Cluster number	Cluster 4	Cluster 1	Cluster 5	Cluster 4
Haddock score	−88.9 ± 6.1	−94.0 ± 2.8	−114.6 ± 6.4	−80.0 ± 6.3
Rmsd from the overall lowest-energy structure	8.5 ± 1.1	12.4 ± 0.1	0.5 ± 0.4	1.1 ± 0.8
Van der Waals energy	−67.7 ± 6.6	−64.8 ± 4.0	−84.0 ± 7.9	−59.7 ± 8.3
Electrostatic energy	−238.4 ± 29.0	−312.5 ± 16.6	−188.7 ± 23.4	−164.5 ± 26.0
Z-score	−1.1	−1.9	−1.6	−2.5

### Determination of IC50 of sericin, dactolisib, and vitamin D against A549 and H460 cells

**Fig. 8:**
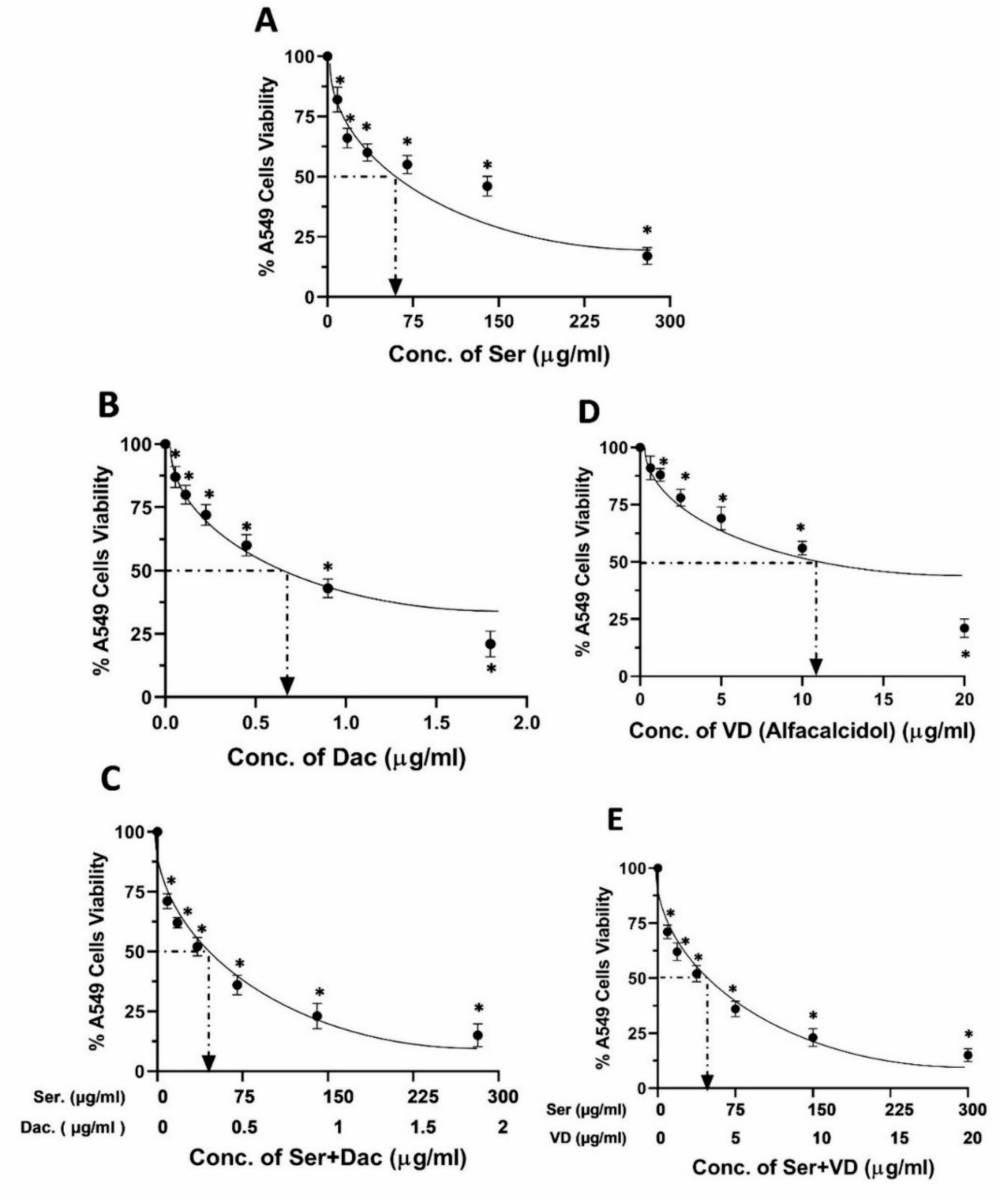
The viability percentages of A549 cells treaded with (**A**) sericin (Ser), (**B**) dactolisib (Dac), (**C**) vitamin D (alfacalcidol) (VD), (**D**) sericin and dactolisib (Ser + Dac), and (**E**) sericin and vitamin D (Ser + VD) using the MTT assay. The MTT assay was carried out in three independent replicates, and all values are depicted as mean ± SD. *p < 0.05 indicates a significant difference for Ser, Dac, and/or VD versus the corresponding control group.

**Fig. 9:**
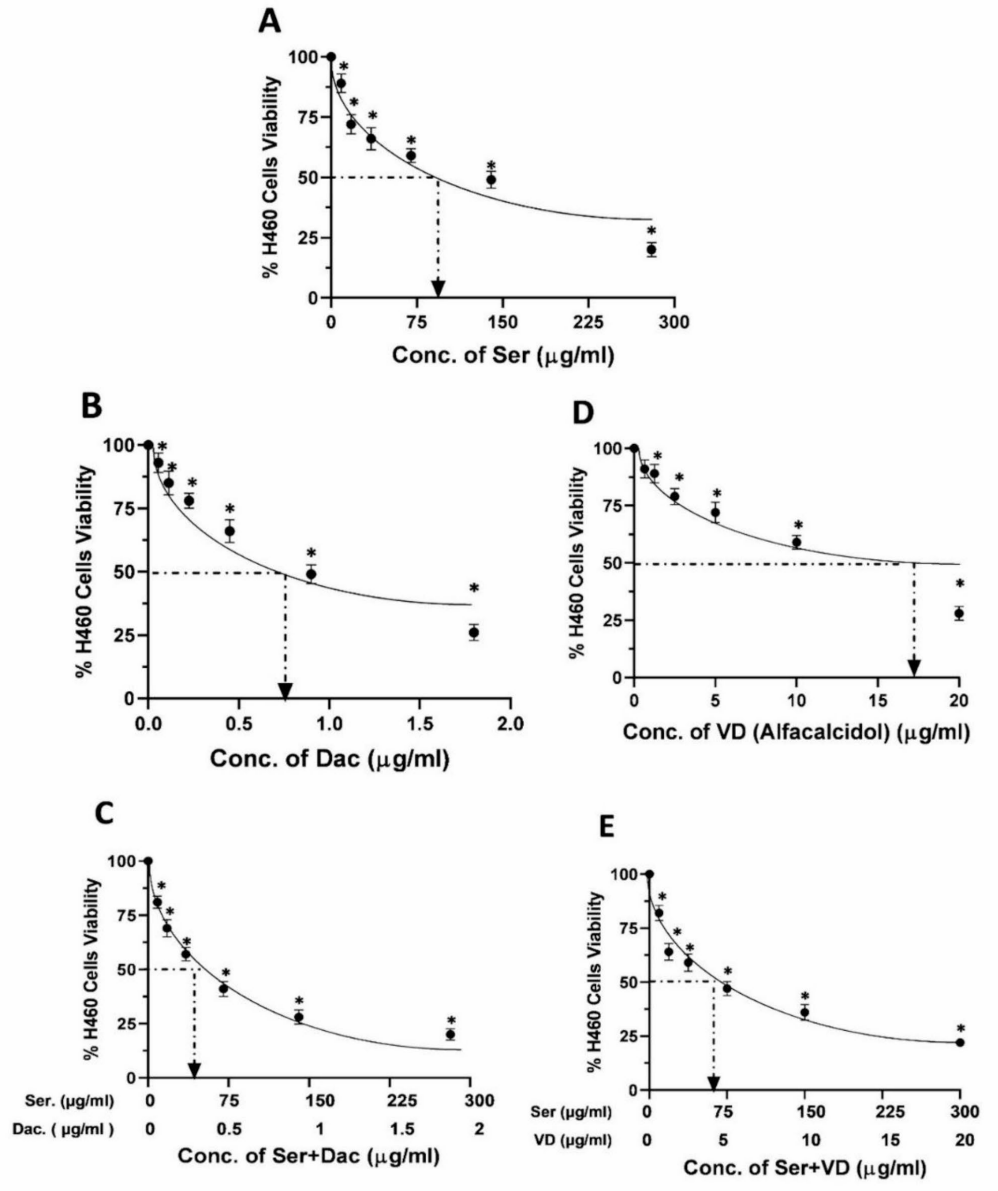
The percentages of H460 cell viability after treatment with (**A**) sericin (Ser), (**B**) dactolisib (Dac), (**C**) vitamin D (alfacalcidol) (VD), (**D**) sericin and dactolisib (Ser + Dac), and (**E**) sericin and vitamin D (Ser + VD) using the MTT assay. The MTT assay was conducted in three independent replicates, and all values are depicted as mean ± SD. *p < 0.05 points to a significant difference for Ser, Dac, and/or VD versus the corresponding control group.

The impacts of sericin, dactolisib, and vitamin D on the viability of both A549 and H460 cells were evaluated, revealing concentration-dependent cytotoxic effects as illustrated in Figs. 8 and 9, respectively. Specifically, cells treated with different concentrations of sericin ranging from 5 to 280 µg/ml hindered the viability of A549 and H460 cells, reporting an IC50 of 62.4 µg/ml and 83 µg/ml, respectively. Similarly, dactolisib at concentrations between 0.05 and 1.8 µg/ml strongly potently impeded cell proliferation, with an IC50 value of 0.6 µg/ml for A549 cells and 0.8 µg/ml for H460 cells. In the same manner, vitamin D concentrations in a range from 0.5 to 20 µg/ml inhibited the viability of A549 and H460 cells with IC_50_ of 11.6 µg/ml and 17.5 µg/ml, respectively. Furthermore, combinatorial treatment of sericin and dactolisib with an identical concentration range precluded cell viability with a combined IC_50_ value of 32.112 µg/ml (31. 9 µg/ml for sericin + 0.212 µg/ml for dactolisib) for A549 cells and an IC_50_ value of 48.214 µg/ml (47.9 µg/ml for sericin + 0.314 µg/ml for dactolisib) for H460 cells. On the other hand, co-treatment with sericin and vitamin D within the similar concentration range exhibited a hindrance to cell viability, recording a combined IC_50_ value of 45.29 µg/ml (41.8 µg/ml for sericin + 3.49 µg/ml for vitamin D) for A549 cells and an IC_50_ value of 59.92 µg/ml (55.3 µg/ml for sericin + 4.62 µg/ml for vitamin D) for H460.

### Evaluation of CIs and DRIs of sericin, dactolisib, and vitamin D toward A549 and H460 cells

To investigate the synergetic influences of sericin, dactolisib, and vitamin D combinations, synergy experiments were conducted against A549 and H460 cells. The cells were treated with sericin, dactolisib, and/or vitamin D, and CompuSyn software was used to figure out the drug interaction of the sericin, dactolisib, and/or vitamin D combination.

Table 7 demonstrates the CIs computed by CompuSyn software at IC_50_ after treating A549 cells with sericin, dactolisib, vitamin D, and their combinations. At IC_50_, the CI value was 0.877 for (Ser + Dac) and 0.972 for (Ser + VD). Importantly, the CI values of both combinations were lower than 1, implying synergistic impacts of the Ser + Dac and Ser + VD combinations versus A549 cells. Likewise, Table 7 presents dose reduction indices analyses that further emphasize the advantageous effects of the running combinations. Adding sericin to dactolisib hampered the growth of A549 cells by 50% at lower doses than those used for each drug alone. Precisely, the DRI values of sericin and dactolisib were reported at 1.95 and 2.73, respectively. In a similar manner, adding sericin to vitamin D induced a remarkable dose reduction of sericin by 1.48 fold and 3.3 fold for VD.

**Table 7:** Cls and DRIs calculated by CompuSyn software to analyze the growth hindrance of A549 cells after treatment with a combination of ser + dac and ser + VD for 48 h.

Drug \Combo	CI value	IC_50_ Dose Ser (µg/ml)	IC_50_ Dose Dac (µg/ml)	IC_50_ Dose VD (µg/ml)	DRI Ser	DRI Dac	DRI VD
Ser		62.4					
Dac			0.6				
VD				11.6			
Ser + Dac	0.877	31.9	0.212		1.95	2.73	
Ser + VD	0.972	41.8		3.49	1.48		3.3

Combination indices (Cls), dose reduction indices (DRIs), sericin (Ser), dactolisib (Dac), and vitamin D (VD).

Considering the CIs of the studied drugs versus H460 cells, it is apparent from the data in Table 8 that the CIs of the combinations at IC_50_ were 0.98 for (Ser + Dac) and 0.93 for (Ser + VD). This indicates the significant synergistic impacts of sericin and dactolisib and sericin and vitamin D combinations against H460 cells. Furthermore, dose reduction indices analyses manifested that adding sericin to dactolisib suppressed the growth of A549 by 50% utilizing a reduced dose than the doses of each drug alone, where DRIs of sericin and dactolisib were reported to be 1.73 and 2.45, respectively, as shown in Table 8. Similarly, adding sericin to vitamin D led to a notable dose reduction of 1.49 fold and 3.79 fold for vitamin D.

**Table 8:** Cls and DRIs calculated by CompuSyn software to evaluate the growth inhibition of H460 cells as a result of treatment with combinations of ser + dac and ser + VD for 48 h.

Drug \Combo	CI value	IC_50_ Dose Ser (µg/ml)	IC_50_ Dose Dac (µg/ml)	IC_50_ Dose VD (µg/ml)	DRI Ser	DRI Dac	DRI VD
Ser		83.0					
Dac			0.8				
VD				17.5			
Ser + Dac	0.98	47.9	0.314		1.73	2.45	
Ser + VD	0.93	55.3		4.62	1.49		3.79

Combination indices (Cls), dose reduction indices (DRIs), sericin (Ser), dactolisib (Dac), and vitamin D (VD).

### Influence of sericin, dactolisib, and vitamin D treatments on protein content in A549 and H460 cells

**Fig. 10:**
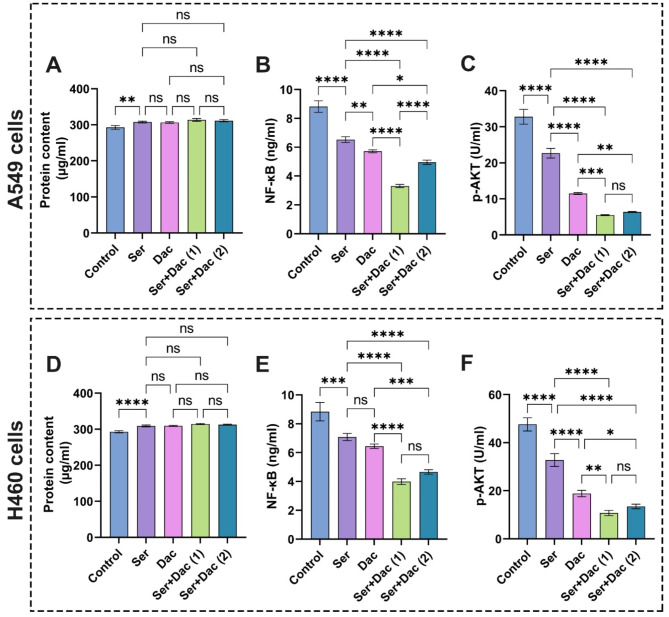
Evaluation of (**A**) and (**D**) protein content, (**B**) and (**E**) NF-кB, (**C**) and (**F**) p-AKT in A549 and H460 lung cancer cell lines after treatment with sericin and dactolisib. The results are shown as mean ± SD when *n* = 6. The analysis was implemented using one-way ANOVA linked with multiple comparisons between different groups following Tukey’s (****p < 0.0001, ***p < 0.001, **p < 0.01, and *p < 0.05, ns denotes non-significant differences). Abbreviations: Sericin (Ser), dactolisib (Dac), Phospho-Akt (p-Akt), and Nuclear factor kappa-light-chain-enhancer of activated B cells (NF-κB). Ser + Dac (1) group: the cells treated with sericin and dactolisib at IC_50_ for each drug, while Ser + Dac (2) group: the cells treated with sericin and dactolisib at IC_50_ following combination indices (CIs).

**Fig. 11:**
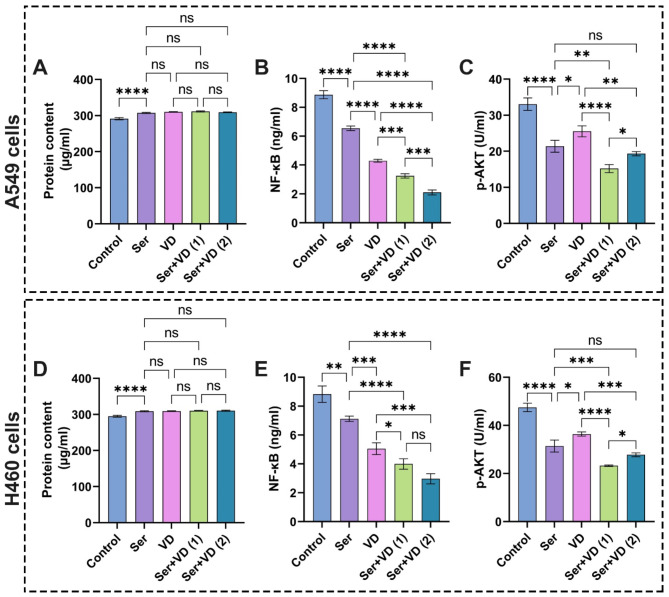
Assessment of (**A**) and (**D**) protein content, (**B**) and (**E**) NF-кB, (**C**) and (**F**) p-AKT in A549 and H460 lung cancer cell lines after treatment with sericin and dactolisib. The results are expressed as mean ± SD when *n* = 6. The analysis was implemented using one-way ANOVA linked with multiple comparisons between different groups following Tukey’s (****p < 0.0001, ***p < 0.001, **p < 0.01, and *p < 0.05, ns denotes non-significant differences). Abbreviations: Sericin (Ser), vitamin D (VD), Phospho-Akt (p-Akt), and Nuclear factor kappa-light-chain-enhancer of activated B cells (NF-κB). Ser + VD (1) group: the cells treated with sericin and vitamin D at IC_50_ for each drug, while Ser + VD (2) group: the cells treated with sericin and vitamin D at IC_50_ following combination indices (CIs).

Indeed, all molecular and cellular pathways lie in proteins and their functions in terms of expressions and their roles in regulating various cellular cascades. Therefore, it has been shown that proteins content is one of the most reliable biomarkers for diagnosing lung cancer^56^. In the current study, we estimated the proteins content to investigate the ability of cells to assimilate the treated drugs, in addition to determining the influence of these drugs on the proteins content in general. Figures 10A,D and 11A,D depict significant rises in protein content in all groups treated with different groups, whether individual drugs or combinatorial doses of two drugs compared to the untreated cells. These outcomes are considered an indicator, which implies that these drugs affected the major cellular and signaling pathways in the studied lung cancer cells.

### Impact of sericin, dactolisib, and vitamin D treatments on NF-кB in A549 and H460 cells

Inflammation is widely acknowledged as a distinguishing characteristic of cancer and is believed to have a crucial impact on the growth and advancement of most malignancies, including those that have no noticeable manifestations of infection or inflammatory conditions^57^. It is well known that the NF-κB plays key functions as a leading regulator of the expression of crucial modulating genes related to the immune system, inflammatory processes, cellular death, and proliferating cells^58^. Due to the dynamic regulation through varied stimuli, NF-κB stimulation could be defined as a mediator of cell homeostasis^59,60^. Concerning the proliferation of cells, it has been demonstrated that NF-κB orchestrates the expression of various cell cycle regulators, including cyclin A, cyclin D1, and cyclin-dependent kinase 6 (CDK6)^58,61^. Stimulation of NF-κB enhances the expression of various target genes, particularly those responsible for the promotion of cell proliferation and impeding cell death^57^. Furthermore, this stimulation of NF-κB is a crucial process that helps safeguard the cell from apoptosis triggered by tumor necrosis factor alpha (TNF-α)^61^. Crucially, these two roles are intricately connected to the development and progression of cancer^61^. Typically, malignant cells exhibit NF-κB combinatorial activation, uncontrolled growth, or resistance to cell death^61,62^. These observations make NF-κB a favorable target for devising various anticancer therapies since it helps minimize the adverse effects on normal cells^57^. Accordingly, several studies have been dedicated to investigating the efficacy of NF-κB inhibitors in the treatment of different types of cancer^63–65^.

It is obvious from the data in Fig. 10B that the treatment of A549 cells with sericin significantly diminished the NF-κB activity compared to the control cells. These results are in line with previous reports, which showed that sericin inhibited LPS-induced inflammation via MyD88/NF-κB pathway^66^. On the other hand, the A549 cells doped with dactolisib showed a substantial decrease in NF-κB activity compared to those exposed to sericin and control cells, which is supported by previous observations^67^. Strikingly, the treatment of A549 cells with the combinatorial therapeutics of sericin and dactolisib at IC_50_ for each drug and IC_50_ according to CI values markedly precluded the activity of NF-κB in comparison with cells treated with a single anticancer agent. Correspondingly, the treatment of H460 cells with single and combinatorial drags revealed the similar performances as shown in Fig. 10E.

With regard to the role of vitamin D in decreasing NF-κB activity, the treatment of both A549 and H460 cells with vitamin D gave rise to significant inhibitions of the NF-κB activity compared to the cells treated with sericin and control cells as illustrated in Fig. 11B,E. These results are consistent with previous findings^68,69^. The cotreatment with sericin and vitamin D heightened their anticancer features, hampering NF-κB activity, implying the synergistic influence of both compounds. Taken together, our results accentuate the synergistic effects of sericin + dactolisib and sericin + VD combinations in promoting cancer cell apoptosis.

### Effect of sericin, dactolisib, and vitamin D treatments on pAKT in A549 and H460 cells

The PI3K/AKT/mTOR signaling pathway is well known to be one of the paramount molecular pathways in cancer disease^70,71^. Specifically, lung cancer can either cause mutations in the AKT genes or unfavorably increase the expression of AKT isoforms^72,73^. Therefore, we appraised the anticancer characteristics of sericin, dactolisib, and vitamin D alongside the respective combinations, including sericin + dactolisib and sericin + vitamin D by estimating the level of the p-AKT produced by lung cancer cells. Figure 10C displays that p-AKT levels were significantly diminished in all treated cells in relation to the control group. As anticipated, the A549 cells exposed to dactolisib at IC_50_ demonstrated a significant lessening in p-AKT levels in comparison with those treated only with sericin. The potency of dactolisib to preclude the PI3K/mTOR signaling pathway and its respective proteins, predominantly p-AKT, could explain this performance, which is similar to previous observations^10,12^. Intriguingly, compared to control cells, the A549 cells cotreated with sericin and dactolisib at IC_50_ for each drug and IC_50_ according to CI values significantly reduced the p-AKT level compared to the dactolisib-treated cells, implying the efficacy of the combination to hinder the emergence of cancer cells resistant to the drugs. Comparable findings could be perceived in the case of H460 cells as shown in Fig. 10F.

With regard to the Ser + VD (sericin + vitamin D) combination, the sericin significantly dropped the p-AKT level compared to the control and VD-treated cells as shown in Fig. 11C,F. On the other hand, the Ser + VD combination regime led to enhancement of their anticancer features compared to the single drug as recognized by the prominent reduction in p-AKT level. Overall, these findings corroborated the synergistic effect of sericin and dactolisib, or sericin and vitamin D, in inhibiting the level of P-AKT, suggesting their competency as PI3K/AKT/mTOR pathway inhibitors.

### Impact of sericin and vitamin D treatments on VDR level in A549 and H460 cells

It has been demonstrated that expression and activation of the VDR are indispensable factors for comprehending the function of vitamin D in addition to explaining the anticancer attributes of various drugs^74,75^. The nuclear vitamin D receptor (VDR) is found on chromosome 12q13.11, spanning approximately 100 kb and consisting of five promoters, eight coding exons, and six untranslated exons^76^. It has been explored that the binding of 1,25-D3 to VDR prompts both genomic and non-genomic governing of downstream targets, which incorporate diverse biological functions, such as anti-differentiation and anti-proliferation activities in cancer cell lines^77^. Besides, previous reports manifested the detection of VDR in association with tuberculosis and cancers, including colorectal cancer and lung cancer^69,78,79^.

**Fig. 12:**
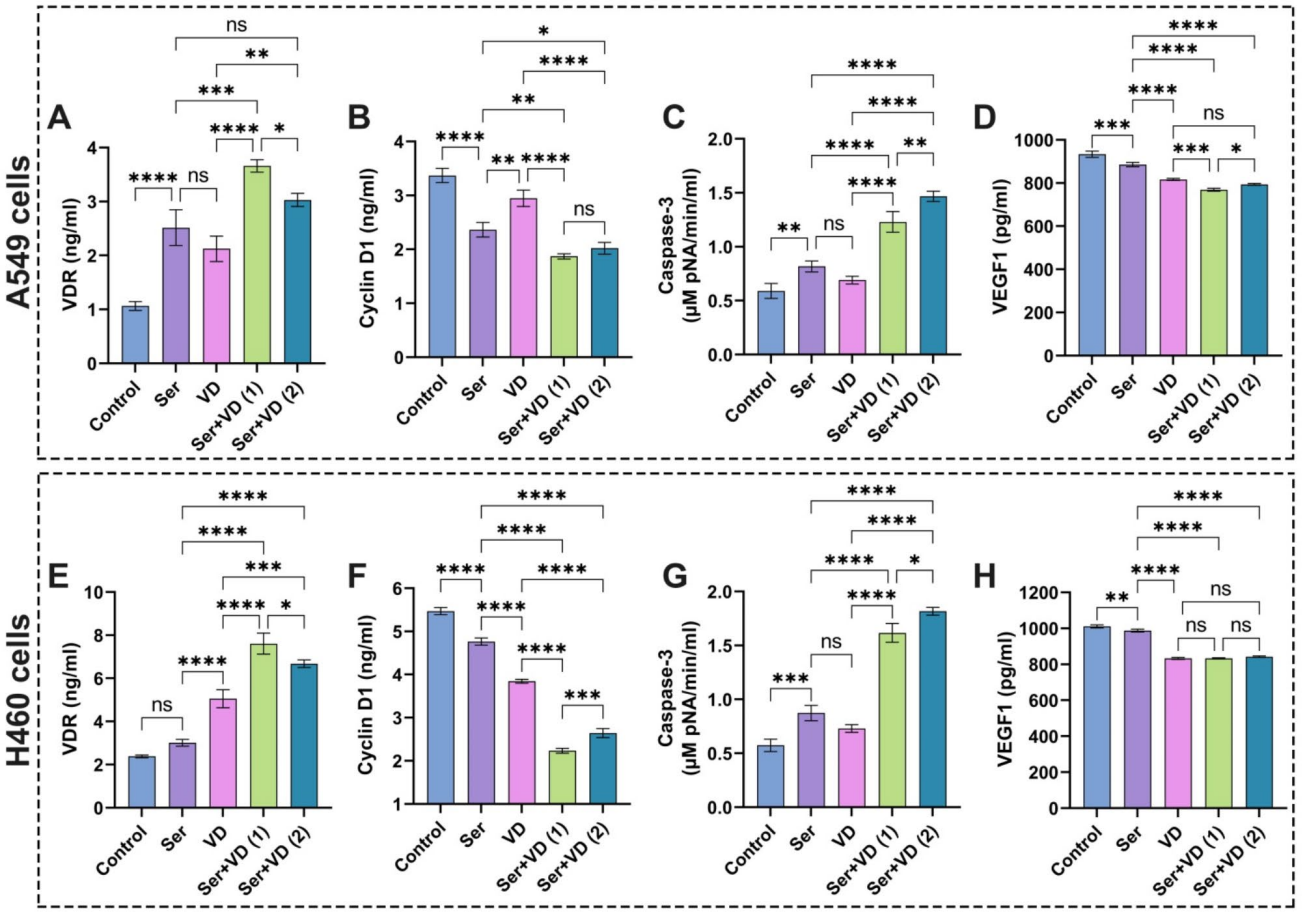
Evaluation of (**A**) and (**E**) VDR, (**B**) and (**F**) cyclin D1, (**C**) and (**G**) caspase-3, and (**D**) and (**H**) VEGF1 in A549 and H460 lung cancer cell lines after treatment with sericin and vitamin D. The results are displayed as mean ± SD when *n* = 6. The analysis was implemented using one-way ANOVA linked with multiple comparisons between different groups following Tukey’s (****p < 0.0001, ***p < 0.001, **p < 0.01, and *p < 0.05, ns denotes non-significant differences). Abbreviations: Sericin (Ser), vitamin D (VD), vitamin D receptor (VDR), cysteine-requiring Aspartate protease (caspase-3), and vascular endothelial growth factor1 (VEGF1). Ser + VD (1) group: the cells treated with sericin and vitamin D at IC_50_ for each drug, whereas Ser + VD (2) group: the cells treated with sericin and vitamin D at IC_50_ following combination indices (CIs).

It could be perceived from the data in Fig. 12A,E that treatment of A549 and H460 cells with sericin resulted in notable elevations of the VDR level, pointing to the anticancer efficiency of sericin, particularly the cells treated with vitamin D exhibited a lower VDR level than sericin. Strikingly, the administration of combinatorial doses of sericin and vitamin D at IC_50_ considerably enhanced the VDR response, which was higher than the combinatorial doses of both drugs at IC_50_ according to CI values. Taken together, these findings propose the capacity of sericin and vitamin D combination to promote their anticancer properties by regulating various metabolic pathways through VDR expression.

### Evaluation of sericin, dactolisib, and vitamin D treatments on cyclin D1 in A549 and H460 cells

D-cyclins, such as cyclins D1, D2, and D3, form active structures that implicate in CDK4 or CDK6. Besides, they predominantly contribute to the progress of cell cycles in terms of extracellular stimulation^80^. Given the role of D cyclins in intermediating extracellular signs of cell proliferation, it is anticipated that the amplification of D cyclins leads to neoplastic evolution. It is of particular interest that cyclin D1 commonly undergoes dysregulation throughout tumorigenesis compared to cyclins D2 or D3^81^; thus, it is necessary to profoundly investigate the expression of cyclin D1 during cancer management.

**Fig. 13:**
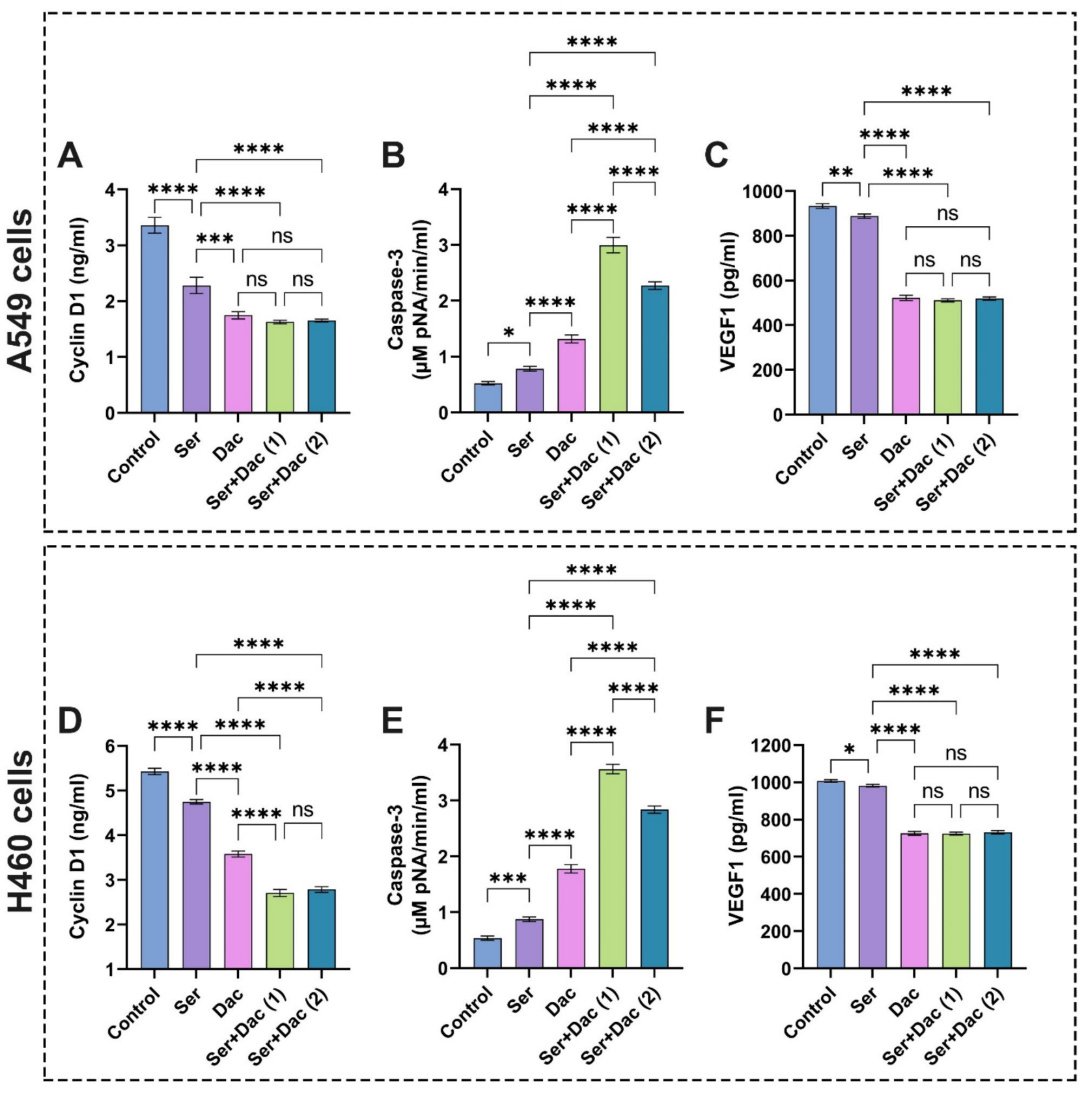
Assessment of (**A**) and (**D**) cyclin D1, (**B**) and (**E**) caspase-3, and (**C**) and (**F**) VEGF1 in A549 and H460 lung cancer cell lines after treatment with sericin and dactolisib. The results are shown as mean ± SD when *n* = 6. The analysis was implemented using one-way ANOVA linked with multiple comparisons between different groups following Tukey’s (****p < 0.0001, ***p < 0.001, **p < 0.01, and *p < 0.05, ns points to non-significant differences. Abbreviations: Sericin (Ser), dactolisib (Dac), cysteine-requiring Aspartate protease (caspase-3), and vascular endothelial growth factor1 (VEGF1). Ser + Dac (1) group: the cells treated with sericin and dactolisib at IC_50_ for each drug, while Ser + Dac (2) group: the cells treated with sericin and dactolisib at IC_50_ following combination indices (CIs).

It could be discernible from the data in Fig. 13A,D that cells treated with sericin remarkably reduced cyclin D1 expression in comparison with control cells. These results correspond with previous reports, which evidenced the expression modification of genes like cyclin-dependent kinase inhibitors after treatment with sericin and sericin-AgNO_3_ NPs^82^. On the other hand, the cells doped with dactolisib showed a notable decrease in cyclin D1 expression compared to the control cells^12^. The cotreatment with sericin and dactolisib at IC_50_ for each drug and IC_50_ following CI values significantly lessened the cyclin D1 level compared to the control cells without significant difference with regard to the cells treated with dactolisib.

On the other hand, vitamin D decreased the levels of cyclin D1 as delineated in Fig. 12B,F. These findings are in accordance with previous investigations, which explained the paramount role of vitamin D in constraining the signal transduction of cancer pathways like cyclin D1, precluding the calcium binding role of HRC (Histidine-Rich Calcium-Binding) protein^83^. The treatment of cells with combinatorial therapy of sericin and vitamin D at IC_50_ for each drug and IC_50_ achieving CI values markedly inhibited cyclin D1 compared to sericin. Overall, the combination of sericin and vitamin D demonstrated stronger activity in inhibiting cyclin D1 expression than sericin and dactolisib.

### Assessment of sericin, dactolisib, and vitamin D treatments on caspase-3 activity in A549 and H460 cells

To evaluate the anti-proliferative potency of the examined drugs, the level of caspase-3 was estimated after treatment with individual and combinatorial drugs. It is widely perceived that casapase-3 plays a pivotal role in modulating apoptosis and triggering the malignancy of various cancers, including hepatocellular carcinoma, prostate cancer, breast cancer, and lung cancer^84,85^.

It is evident from the data in Fig. 13B that the treatment of A549 cells with sericin remarkably expanded the caspase-3 activity compared to the control cells, implying its efficiency to induce the apoptosis of cells. These results are in line with previous reports, which evidenced that sericin activated the extrinsic apoptosis pathway by the caspase cascade CASP8/10 and CASP3/7^25^. On the other hand, the A549 cells doped with dactolisib revealed a significant rise in caspase-3 activity compared to those exposed to sericin and control cells. These findings are consistent with a previous study^12^. Strikingly, the cotreatment of A549 cells with sericin and dactolisib at IC_50_ for each drug and IC_50_ according to CI values markedly amplified the activity of caspase-3 in comparison with cells treated with each individual drug. Importantly, similar results could be observed in H460 cells, corroborating the efficiency of the examined anticancer agents as displayed in Fig. 13E. These findings point out that the addition of sericin to dactolisib potentiated their anticancer properties by promoting the apoptosis of lung cells.

Considering the anti-proliferative activity of sericin and vitamin D in relation to A549, H460 cells, Fig. 12C,G illustrate that treatment with sericin or vitamin D substantially elevated the activity of caspase-3 compared to the control cells without significant difference between both drugs. These findings are correlated with previous investigations, which demonstrated the anti-cancer potential of vitamin D3^86^. On the other hand, cotreating cells with sericin and vitamin D at IC_50_ for each compound and IC_50_ attaining CI values resulted in a significant increase in caspase-3 activity compared to those treated alone with sericin or VD. Overall, our findings clearly indicate the synergistic influences of combinations of sericin + dactolisib and sericin + vitamin D in enhancing the apoptosis of cancer cells.

### Appraisal of sericin, dactolisib, and vitamin D treatments on VEGF1 level in A549 and H460 cells

It is recognized that the VEGF plays a crucial role in sustaining the development and progression of lung cancer by promoting the proliferation of endothelial cells, boosting vascular permeability, and activating endothelial cell migration^2^. Raised VEGF expressions were determined in diverse tumor cells, including breast and prostate cancers, colorectal, stomach, pancreatic, and lung cancer, particularly NSCLC^2,87^. Therefore, it is considered a key indicator in examining the development of lung cancer and appraising the efficacy of various therapies in hampering lung cancer^2,88^.

It is apparent from the data in Fig. 13C,F that the treatment of lung cancer cells A549 and H460 cells with sericin significantly diminished the VEGF1 expression compared to the control cells. These findings are in line with previous reports, which evidenced the potency of anticancer peptides to decrease VEGF1 levels^2^. The anticancer properties of sericin could be related to its efficiency to instigate apoptotic pathways, impede angiogenesis, and activate cell cycle arrest^89^. Remarkably, the cells doped with dactolisib exhibited a significant lessening in VEGF1 levels compared to those treated with sericin and control cells. These results match previous investigations against prostate cancer due to the efficacy of dactolisib to thwart the phosphoinositide-3-kinase/mechanistic target of rapamycin (PI3K/mTOR) signaling pathway to curb unsusceptible antitumor activity^10,12^. On the other hand, the treatment of cells with combinatorial therapy of sericin and dactolisib at IC_50_ for each drug and IC_50_ following CI values substantially reduced the VEGF1 level compared to the control cells without significant difference in relation to the dactolisib treatment. However, the application of dactolisib alone could engender disease stagnation^10^; therefore, the presence of sericin could promote the effectiveness and stability of the drug in addition to decreasing its side effects on normal cells.

Figure 12D,H depict that the vitamin D significantly lowered the VEGF1 level compared to sericin and the control groups. These findings are consistent with previous reports^83^. This behavior could be explained by the pivotal role of vitamin D in modulating whole tumorigenesis mechanisms, including proliferation, apoptosis, autophagy, and angiogenesis of cells^90,91^. The addition of sericin to vitamin D boosted their anticancer features, enhancing the decrease in VEGF1 level compared to each drug alone. Altogether, the combination of sericin and dactolisib emphasized prominent activity in inhibiting VEGF1 expression compared to sericin and vitamin D.

## Conclusion

To sum up, the combination of sericin + dactolisib and sericin + vitamin D exhibited notable anticancer activities at low doses against non-small lung cancer cells (A549 and H460 cells). The computational investigations exhibited the outstanding affinities of both combinations with NF-κB, Cyclin D1, p-AKT, and VEGF1 proteins. Intriguingly, in vitro results demonstrated a striking correlation with in silico examinations, showing decreased levels of NF-κB, Cyclin D1, p-AKT, and VEGF1 associated with significant promotion of lung cancer cell apoptosis. Altogether, our findings provide compelling evidence for the anticancer properties of sericin combined with dactolisib or vitamin D against non-small lung cancer cells, which serve as a base for future investigations.

## Electronic supplementary material

Below is the link to the electronic supplementary material.

Supplementary Material 1

## Data Availability

The datasets used and/or analyzed during the current study are available from the corresponding author on reasonable request.
