# Supplementary material for: Repurposing of sericin combined with dactolisib or vitamin D to combat non-small lung cancer cells through computational and biological investigations
Source: Sci Rep. 2024 Nov 7;14:27034. doi: 10.1038/s41598-024-76947-0 (PMC11541877; doi:10.1038/s41598-024-76947-0)
Supplement: Supplementary file 1 — Supplementary Material 1 [file 41598_2024_76947_MOESM1_ESM.docx]

**Repurposing of sericin combined with dactolisib or vitamin D to combat non-small lung cancer cells through computational and biological investigations**

**Maged W. Helmy^1^, Mariam H. Youssef^2^, Imane Yamari^3^, Alaa Amr^2^, Farouzia I. Moussa^2^, Abeer El Wakil^4^, Samir Chtita^3^, Lamia M. El-Samad^2,*^, Mohamed A. Hassan^5,*^**

^1^Pharmacology and Toxicology Department, Faculty of Pharmacy, Damanhour University, Postal code: 22511, Egypt.

^2^Department of Zoology, Faculty of Science, Alexandria University, Egypt.

^3^Laboratory of Analytical and Molecular Chemistry, Faculty of Sciences Ben M’Sik, Hassan II University of Casablanca, P. O. Box 7955, Casablanca, Morocco.

^4^Department of Biological and Geological Sciences, Faculty of Education, Alexandria University, Alexandria, Egypt.

^5^Protein Research Department, Genetic Engineering and Biotechnology Research Institute (GEBRI), City of Scientific Research and Technological Applications (SRTA-City), New Borg El-Arab City, 21934 Alexandria, Egypt.

***Corresponding authors**

**1. Lamia M. El-Samad Email: lamya.moustafa@alexu.edu.eg**

**2. Mohamed A. Hassan** **Email: madel@srtacity.sci.eg**

**Table S1.** Kits used in this study to estimate different biological parameters

| **Kit** | **Cat. No.** | **Manufacturer** |
| --- | --- | --- |
| Pierce® BCA protein assay | 23227 | Thermo Scientific, USA |
| Phospho-Akt (p-Akt) ELISA | EIA-3996 | DRG International, Inc., Massachusetts, USA |
| Phospho-NF-κB p65 (S536) ELISA | PEL-NFKBP65-S536 | Ray Biotech, Georgia, USA |
| Cyclin D1 (CD1) ELISA | MBS724349 | MyBioSource, CA, USA |
| Vascular endothelial growth factor (VEGF) | CSB-E11718h | CUSABIO, Maryland, USA |
| Cysteine-requiring Aspartate protease (caspase-3 activity) assay | ab39401 | Abcam Co., Berlin, Germany |

**Table S2.** Non-covalent interaction for the different studied complexes

| Complex | Residues in receptor | Residues in ligand (sericin) | Type of interaction | Distances (angstron) |
| --- | --- | --- | --- | --- |
| Sericin-NF-κB | ARG408  GLY409  PHE411  LYS482  LYS517  GLY558  GLU560  ARG601  GLY602 | ASN106  HIS92  THR94  ASN106  ASP78  ASN64  ASN64  THR63  SER47 | Hydrogen bond | 2.80  3.22  2.64  3.13  2.69  2.71  3.04  2.73  2.99 |
|  | GLY407  ARG408  ARG408  ARG408  ARG408  GLY409  GLY409  SER410  PHE411  PHE411  PHE411  PHE411  PHE411  PHE411  PHE411  GLY412  GLN479  LYS482  LYS482  LYS517  LYS517  PRO557  PRO557  GLY558  GLY558  THR559  GLU560  GLU560  GLU560  ARG601 | ASN106  ASN106  SER105  HIS107  ASP118  ASN106  HIS92  ASP78  HIS92  THR94  ASP78  ASN80  ASN64  ILE66  ASN79  THR94  ASN106  SER105  ASN106  ASP78  HIS92  ASP50  ASN64  ASN64  ASP50  ASN64  ASN64  THR63  ASN49  SER47 | Non-bonded contacts | 3.68  3.83  3.37  3.88  3.64  3.56  3.40  3.51  3.85  3.55  3.66  3.90  3.66  3.73  3.74  3.80  3.46  3.76  3.13  3.49  3.86  3.24  3.12  3.37  3.52  3.30  3.04  3.78  3.65  3.84 |
|  | ARG601  ARG601  GLY602  GLY602  GLY602  PRO603  PRO603  LEU604  CYS605  CYS605  LEU606  LEU606 | GLY62  THR63  SER47  SER32  ASN49  ASN49  ASP34  ASN49  ASP34  ASN35  ASP34  SER19 |  | 3.62  3.77  2.99  3.49  3.57  3.74  3.69  3.85  3.86  3.72  3.41  3.27 |
|  | ARG408  LYS517 | ASP118  ASP78 | Salt bridges | 3.64  2.69 |
| Sericin-Cyclin D1 | ASP34  ASN35  ASP50  THR63  ASN64  ASP78  ASN106  SER110  LYS114 | TYR227  TYR226  ARG228  ARG231  ARG231  ARG235  ARG26  GLU11  THR12 | Hydrogen bonds | 2.65  3.17  2.58  3.00  2.74  2.71  2.74  3.22  2.69 |
|  | VAL22  ASP34  ASN35  ASN35  ASN49  ASP50  ASP50  ASP50  ASP50  THR63  THR63  ASN64  ASN64  ILE66  ASN77  ASP78  ASP78  ASP78  ASN80  HIS92  HIS92  THR94  HIS107  VAL108  VAL108  SER110  SER110  SER110  LYS114  LYS114 | ASN221  TYR227  TYR227  TYR226  ARG228  TYR227  ARG228  ARG231  ARG228  ARG228  ARG231  PHE232  ARG231  LYS167  ARG235  ARG235  PHE232  ARG235  LYS167  LYS167  ARG235  ASP25  ARGG26  ILE13  ASP25  GLU11  CYS8  ILE13  GLU11  THR12 | Non-bonded contacts | 3.63  3.77  3.61  3.82  3.85  3.86  3.47  3.78  3.38  3.72  3.16  3.78  2.74  3.58  3.12  3.83  3.23  3.79  3.30  3.79  3.69  3.38  3.46  3.73  3.40  3.22  3.75  3.33  3.83  3.79 |
|  | ASP50  ASP78  LYS114 | ARG228  ARG235  GLU11 | Salt bridges | 2.58  2.71  2.79 |
| Sericin-p-Akt | ASP50  ASP50  ASP50  THR52  THR63  ASN64  THR68  ASP69  ASP78  ASN80  SER105  THR117  ASP118 | LYS154  TYR152  LEU155  LYS154  LYS158  LEU155  ARG241  ARG241  LYS158  GLU234  LYS297  HIS354  LYS297 | Hydrogen bonds | 2.81  2.85  2.98  3.25  3.07  2.95  2.99  2.61  2.66  2.90  2.96  2.73  2.64 |
|  | ASP50  ASP50  ASP50  THR52  ASN64  ASN64  ASN64  THR68  ASP69  ASP78  ASP78  ASN80  ASN80  HIS92  SER105  THR117  ASP118  ASP118  ASP50  ASP69  ASP78  ASP118  ASP50  ASP50 | LYS154  TYR152  LEU155  LYS154  LEU155  LYS158  LYS154  ARG241  ARG241  LYS158  LEU155  PHE237  GLU234  LEU156  LYS297  HIS354  HIS354  LYS297  LYS154  ARG241  LYS158  LYS297  LYS154  TYR152 | Non-bonded contacts | 3.35  3.85  2.98  3.25  3.80  3.50  3.63  3.87  3.86  3.66  3.67  3.80  3.84  3.52  3.54  3.84  3.73  3.06  3.27  2.61  2.66  2.64  3.35  3.85 |
|  | THR52  ASN64  ASN64  THR68  ASP69  ASP78  ASP78  ASN80  ASN80  HIS92  SER105  THR117  ASP118  ASP118 | LYS154  LEU155  LYS158  ARG241  ARG241  LYS158  LEU155  PHE237  GLU234  LEU156  LYS297  HIS354  HIS354  LYS297 |  | 3.40  2.98  3.50  3.87  3.86  3.66  3.22  3.80  3.84  3.52  3.54  3.84  3.73  3.06 |
|  | ASP50  ASP69  ASP78  ASP118 | LYS154  ARG241  LYS158  LYS297 | Salt bridges | 3.27  2.61  2.66  2.64 |
| Sericin-VEGF1 | SER19  SERE47  ASN49  ASP50  THR63  HIS92 | LEU32  GLY59  GLY58  ARG23  CYS60  VAL15 | Hydrogen bonds | 2.84  2.65  2.75  2.67  3.14  2.82 |
|  | SER19  SER32  SER47  ASN49  ASP50  SER61  THR63  HIS92 | LEU32  CYS57  GLY59  GLY58  ARG23  GLU64  SER24  VAL15 | Non-bonded contacts | 2.84  3.50  2.65  2.75  2.67  3.27  3.70  2.82 |
|  | ASP50 | ARG23 | Salt bridges | 2.67 |


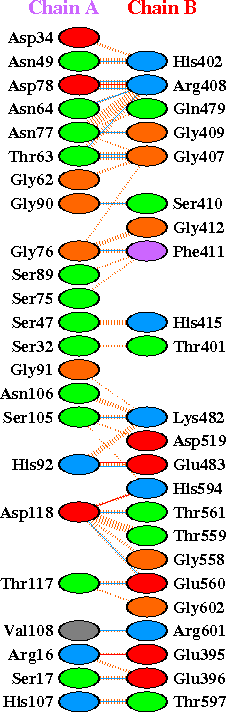


|  |  | \|  \| **Key:** \| 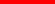 \|  \| Salt bridges \|  \| 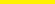 \|  \| Disulphide bonds \|  \| 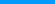 \|  \| Hydrogen bonds \|  \| 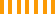 \|  \| Non-bonded contacts \| \| --- \| --- \| --- \| --- \| --- \| --- \| --- \| --- \| --- \| --- \| --- \| --- \| --- \| --- \| --- \| --- \| --- \| \|  \|  \| \| \| \| \| \| \| \| \| \| \| \| \| \| \| \|    |  |
| --- | --- | --- | --- | --- | --- | --- | --- | --- | --- | --- | --- | --- | --- | --- | --- | --- | --- | --- | --- | --- | --- | --- | --- | --- | --- | --- | --- | --- | --- | --- | --- | --- | --- | --- | --- | --- | --- |

**Fig. S1.** Sericin+Doctalisib (Chain A)-NF-κB (Chain B), interacted residues through the interface.

|  |  | |  |
| --- | --- | --- | --- |
| 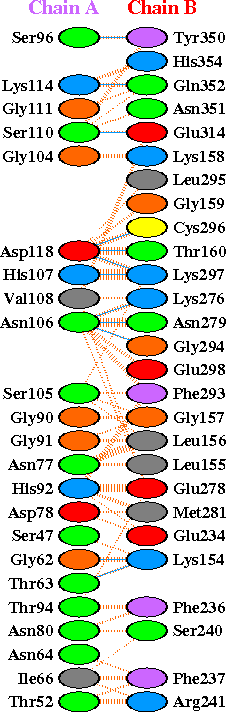 | | | |
| \|  \| **Key:** \| 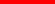 \|  \| Salt bridges \|  \| 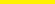 \|  \| Disulphide bonds \|  \| 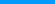 \|  \| Hydrogen bonds \|  \| 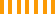 \|  \| Non-bonded contacts \| \| --- \| --- \| --- \| --- \| --- \| --- \| --- \| --- \| --- \| --- \| --- \| --- \| --- \| --- \| --- \| --- \| --- \|    |  |  |  |

**Fig. S2.** Sericin+Doctalisib (Chain A)-Cyclin D1 (Chain B), interacted residues through the interfaces.

|  |  | |  |
| --- | --- | --- | --- |
|  | | | |
| 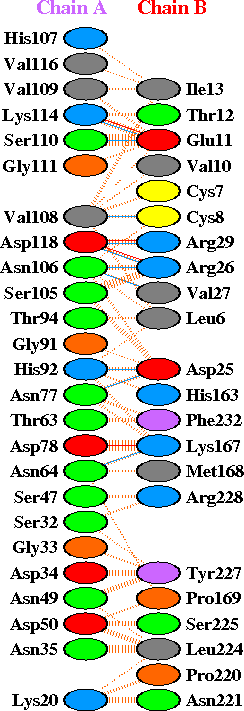 |  |  |  |

|  | **Key:** | 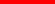 |  | Salt bridges |  | 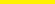 |  | Disulphide bonds |  | 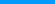 |  | Hydrogen bonds |  | 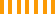 |  | Non-bonded contacts |
| --- | --- | --- | --- | --- | --- | --- | --- | --- | --- | --- | --- | --- | --- | --- | --- | --- |

**Fig. S3.** Sericin+Doctalisib(chain A)–p-AKT (Chain B), interacted residues through the interface.

|  |  | |  |
| --- | --- | --- | --- |
|  | | | |
| 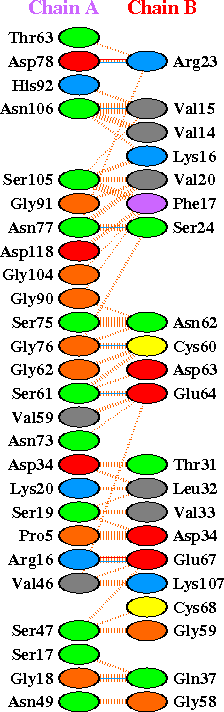 |  |  |  |

|  | **Key:** | 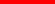 |  | Salt bridges |  | 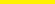 |  | Disulphide bonds |  | 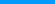 |  | Hydrogen bonds |  | 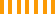 |  | Non-bonded contacts |
| --- | --- | --- | --- | --- | --- | --- | --- | --- | --- | --- | --- | --- | --- | --- | --- | --- |

**Fig. S4.** Sericin+Doctalisib (chain A)-VEGF1 (Chain B), interacted residues through the interfaces.

|  | | | |
| --- | --- | --- | --- |
| 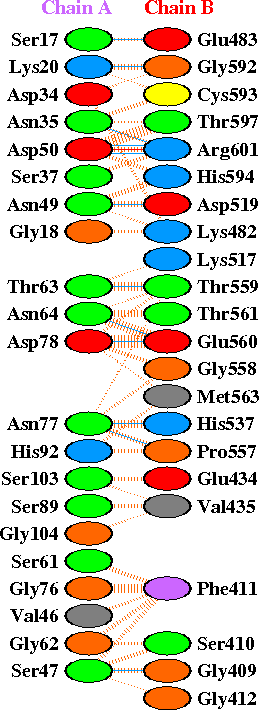 |  |  |  |

|  | **Key:** | 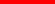 |  | Salt bridges |  | 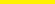 |  | Disulphide bonds |  | 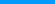 |  | Hydrogen bonds |  | 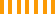 |  | Non-bonded contacts |
| --- | --- | --- | --- | --- | --- | --- | --- | --- | --- | --- | --- | --- | --- | --- | --- | --- |

**Fig. S5.** Sericin+Vitamin D (Chain A)-NF-κB (Chain B), interacted residues through the interfaces.

|  | | | |
| --- | --- | --- | --- |
| 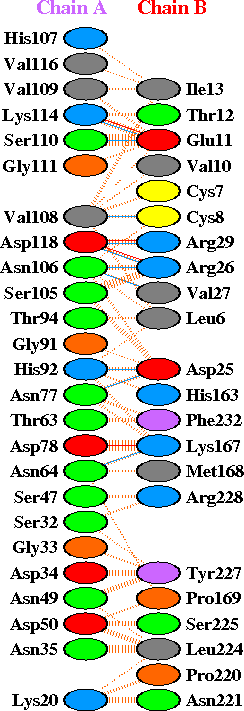 |  |  |  |

|  | **Key:** | 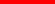 |  | Salt bridges |  | 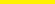 |  | Disulphide bonds |  | 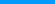 |  | Hydrogen bonds |  | 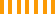 |  | Non-bonded contacts |
| --- | --- | --- | --- | --- | --- | --- | --- | --- | --- | --- | --- | --- | --- | --- | --- | --- |

**Fig. S6.** Sericin+Vitamin D (Chain A)-Cyclin D1 (Chain B), interacted residues through the interfaces.

|  | | | |
| --- | --- | --- | --- |
| 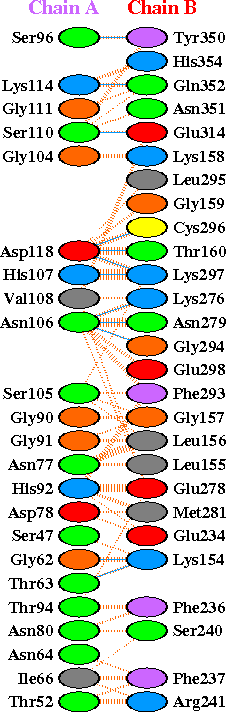 |  |  |  |

|  | **Key:** | 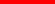 |  | Salt bridges |  | 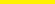 |  | Disulphide bonds |  | 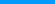 |  | Hydrogen bonds |  | 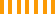 |  | Non-bonded contacts |
| --- | --- | --- | --- | --- | --- | --- | --- | --- | --- | --- | --- | --- | --- | --- | --- | --- |

**Fig. S7.** Sericin+Vitamin D (Chain A)-p-AKT (Chain B), interacted residues through the interfaces.

|  | | | |
| --- | --- | --- | --- |
| 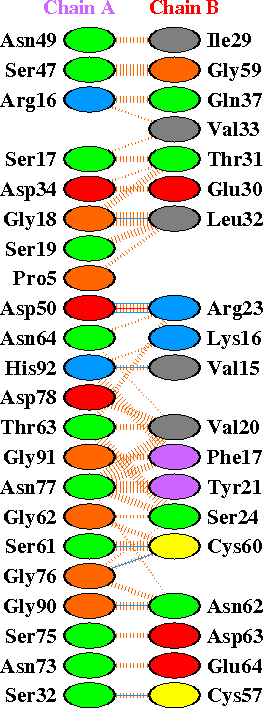 |  |  |  |

|  | **Key:** | 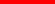 |  | Salt bridges |  | 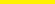 |  | Disulphide bonds |  | 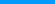 |  | Hydrogen bonds |  | 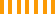 |  | Non-bonded contacts |
| --- | --- | --- | --- | --- | --- | --- | --- | --- | --- | --- | --- | --- | --- | --- | --- | --- |

**Fig. S8.** Sericin+Vitamin D (Chain A)-VEGF1 (Chain B), interacted residues through the interfaces.
